# An Adaptive Physical Activity Intervention for Overweight Adults: A Randomized Controlled Trial

**DOI:** 10.1371/journal.pone.0082901

**Published:** 2013-12-09

**Authors:** Marc A. Adams, James F. Sallis, Gregory J. Norman, Melbourne F. Hovell, Eric B. Hekler, Elyse Perata

**Affiliations:** 1 School of Nutrition and Health Promotion, Arizona State University, Phoenix, Arizona, United States of America; 2 Department of Family and Preventive Medicine, University of California San Diego, San Diego, California, United States of America; 3 Graduate School of Public Health, San Diego State University, San Diego, California, United States of America; 4 Department of Psychology, San Diego State University, San Diego, California, United States of America; 5 College of Education and Allied Studies, California State University East Bay, Hayward, California, United States of America; University of Sao Paulo, Brazil

## Abstract

**Background:**

Physical activity (PA) interventions typically include components or doses that are static across participants. Adaptive interventions are dynamic; components or doses change in response to short-term variations in participant's performance. Emerging theory and technologies make adaptive goal setting and feedback interventions feasible.

**Objective:**

To test an adaptive intervention for PA based on Operant and Behavior Economic principles and a percentile-based algorithm. The adaptive intervention was hypothesized to result in greater increases in steps per day than the static intervention.

**Methods:**

Participants (N = 20) were randomized to one of two 6-month treatments: 1) static intervention (SI) or 2) adaptive intervention (AI). Inactive overweight adults (85% women, M = 36.9±9.2 years, 35% non-white) in both groups received a pedometer, email and text message communication, brief health information, and biweekly motivational prompts. The AI group received daily step goals that adjusted up and down based on the percentile-rank algorithm and micro-incentives for goal attainment. This algorithm adjusted goals based on a moving window; an approach that responded to each individual's performance and ensured goals were always challenging but within participants' abilities. The SI group received a static 10,000 steps/day goal with incentives linked to uploading the pedometer's data.

**Results:**

A random-effects repeated-measures model accounted for 180 repeated measures and autocorrelation. After adjusting for covariates, the treatment phase showed greater steps/day relative to the baseline phase (p<.001) and a group by study phase interaction was observed (p = .017). The SI group increased by 1,598 steps/day on average between baseline and treatment while the AI group increased by 2,728 steps/day on average between baseline and treatment; a significant between-group difference of 1,130 steps/day (Cohen's d = .74).

**Conclusions:**

The adaptive intervention outperformed the static intervention for increasing PA. The adaptive goal and feedback algorithm is a “behavior change technology” that could be incorporated into mHealth technologies and scaled to reach large populations.

**Trial Registration:**

ClinicalTrials.gov NCT01793064

## Introduction

Public health recommendations specify minimum frequency, duration or intensity of physical activity needed for health benefits. For example, 150 minutes of moderate or 75 minutes of vigorous physical activity [1] or 8,000–10,000 steps per day for adults [2]. However, behavior change is not a threshold or linear process. Inactive individuals rarely have the motivation or the fitness needed to attain and then continuously maintain higher physical activity levels on a daily basis. Health behavior theories and derived strategies should account for this complexity, yet many programs currently prescribe minimum amounts to participants (e.g. 10,000 steps/day) or set goals that increase linearly by some fixed amount over the course of an intervention (e.g. 250 steps/week, henceforth we label these “static” interventions) [3].

Adaptive interventions [4], [5] may hold promise for promoting initiation and maintenance of behavior change because behavior is inherently variable [6], [7]. Theoretically, variability is due to changing social and environmental contexts (e.g., childcare, infections, seasons, neighborhood design) [8]–[10]. Static interventions do not account for within-person variability and may be less effective than adaptive ones. Compared to static interventions for physical activity, adaptive treatments have time-varying and performance-based components. For example, if an individual's typical level of physical activity was 3,000 steps per day, it is plausible to adjust the recommended dosage down from 10,000 to 4,000 steps; a dosage that is potentially more feasible for the individual to attain and therefore more likely to support an individual's behavior change. Further, linearly increasing goals (250 steps/week), although they may consider an individual's baseline activity, typically do not consider life events (e.g., sickness, vacations, natural variability in motivation or ability) that sometimes result in lapses of engagement. In a linear approach, these lapses are typically not accounted for in a person's goals thereby creating an artificially high bar for success. Moreover, individuals are likely to have different rates of change (i.e., trajectories) over the course of an intervention. Some individuals respond quickly whereas others respond slowly. A process that adapts the intervention to the individual's performance provides new opportunities for reducing treatment mismatch [4]. Adaptive interventions could increase the likelihood that an intervention is appropriate to a given individual's changing context, potentially increasing intervention adherence and effectiveness [4].

Adaptive interventions also offer new opportunities for taking advantage of principles of behavior. A Behavioral Economic approach [7], [10]–[13] incorporating principles of Operant shaping [14], [15] can be used in eHealth and mobile health (mHealth) technologies to increase physical activity through adaptive goal setting and shaping. Shaping is the process of identifying a final behavioral outcome and slowly moving participants towards that outcome by reinforcing behaviors that are closer and closer approximations to the final outcome. While shaping is a common technique, the application of shaping can differ in quality between and within studies [16]. At worst, feedback is applied inconsistently, non-contingently, or with long delays after desired responses [17]. At best, shaping has been described as an art without formalized rules (e.g. a good coach or teacher) [16]. The use of formalized rules of shaping that can be applied consistently across participants (i.e. algorithm) would allow researchers and interventionists to design continuously adaptive behavioral interventions. As participants attain smaller physical activity goals, earn encouraging feedback, and improve their fitness, they are expected to experience a reduction in perceived barriers and improved efficacy [18].

Pedometers and other physical activity sensors are increasingly being linked to technologies (e.g. websites, mobile phones) and provide unique opportunities for the delivery of adaptive interventions. Pedometers have been used among healthy and chronic disease populations, including overweight populations [19]–[24]. Taking 3,000 to 4,000 steps/day “over and above” routine activities (i.e. 6,000 – 7,000 steps) approximates 30-minutes/day of moderate-to-vigorous intensity physical activity for adults and is consistent with the recommendation to take 8,000–10,000 steps/day [25]. In a JAMA systematic review, Bravata et al. found interventions using a pedometer resulted in a 2,007 steps/day increase (95% CI 878–3,129) compared to controls. Bravata et al. noted that the strongest predictor of improvement was to accomplish a step goal, but an evaluation of goal types was not possible due to only 2 studies reporting information about the number of people who met their goals by any type. Prior studies have used a variety of goals including: asking participants to set goals [22], [26]–[31], prescribing goals for participants by adding a set number of steps to baseline (e.g. 250 steps/day increase each week) [3], [32],_ENREF_19 or providing a fixed 10,000 steps/day goal [33], [34]. The benefits of one goal type over another remain to be determined empirically [20].

The present study developed a theory-based, adaptive physical activity intervention (i.e. adaptive goals and feedback), and tested systematically changing goals and feedback contingencies among inactive overweight adults by comparing it to a pedometer intervention with static goals and feedback. The adaptive intervention assumed within-person variance in physical activity and harnessed that variance to adjust individuals' goals and feedback over time [10]. Few behavioral interventions have approached lifestyle change engineering from this theoretical perspective [35]. We hypothesized that the adaptive intervention would result in more physical activity goals met and greater volume of physical activity (i.e. steps/day) compared to the static intervention.

## Methods

### Ethics Statement

San Diego State University and Arizona State University Institutional Review Boards approved the study, and participants provided written informed consent. The protocol for this trial and supporting CONSORT checklist are available as supporting information; see Checklist S1 and Protocol S1. The ClinicalTrials.gov registry number is NCT01793064 (www.clinicaltrials.gov/ct2/show/NCT01793064).

### Inclusion criteria

Individuals were recruited via electronic announcements and physical flyers. Recruitment materials were posted in multiple settings including coffee shops, local universities and colleges, and on local university staff electronic listservs and electronic public websites (e.g. craigslist). Women and men were eligible for the study if they were between 18 and 65 years old, inactive (less than 1000 metabolic equivalent of task (MET)-minutes/week reported on International Physical Activity Questionnaire (IPAQ)) and overweight (body mass index ≥25). This MET-minute threshold was used to account for individuals' tendency to over-report their activity on the IPAQ. Individuals were excluded if they: had a BMI >45, were unable to walk unassisted, had a medical condition (assessed by Physical Activity Readiness Questionnaire (PAR-Q)) [36], pregnant, using pharmaceuticals (except birth control), currently participating in a commercial or research-related diet or exercise program, planned to leave the county of San Diego, California for more than 10 days over the following 6 months, could not speak and read English, or did not have computer and internet access daily.

Qualifying individuals were invited to the research office for an orientation visit. These individuals were provided with a detailed description of study, completed informed consent procedures, completed surveys, and shown how to use the pedometer. Participants were asked to continue their normal routine over the next 10 days wearing a sealed pedometer that masked their steps. This 10-day run-in phase allowed for participant reactivity to the pedometer to subside and an objective baseline physical activity level to be measured. It also ensured participants were comfortable wearing the pedometer and had the technical capacity to upload their pedometer to the Microsoft's HealthVault website at the end of the run-in period. Participants received $15 for attending the orientation visit.

Participants who uploaded their steps successfully (N = 20) were randomly assigned in sequential order to one of two 6-month physical activity interventions: 1) Static Intervention (SI), or 2) Adaptive Intervention (AI) intervention. A 1∶1 random allocation was determined by the first author using a computer generated random number sequence. Participants and investigators were not blinded to intervention assignment and no adverse events were reported during the trial. This sample size for this pilot study was determined to be financial feasible. The last participant completed the intervention in October 2011.

### Intervention Components

#### Target Behavior

The ultimate target for both groups was walking 10,000 steps/day on five or more days per week. We did not expect all participants to reach this level of activity, but the target was provided as a common long-term goal. Walking was selected as a target behavior because it is a common, free, easy and safe form of activity with known health benefits [37]. A target of 8,000–10,000 steps/day approximates the national aerobic moderate-to-vigorous physical activity guideline when steps from routine activities are counted [38].

#### Communication Mediums

Communication with all participants was conducted via brief emails and text messages. These components represented the “front end” of the intervention for participants. Regardless of the medium, all planned communication was designed to be ≤160 characters.

#### Pedometer and Self-monitoring

Participants in both groups were equipped with the Omron HJ-720ITC pedometer during orientation. The Omron has a 7-day LCD display and 41-day internal memory. Participants in both groups used the pedometer un-blinded throughout the 170-day intervention phase.

#### Brief Health Information

During the first week of intervention, participants in both groups were sent via email two brochures on physical activity. One published by the U.S. Health and Human Services was entitled, “Be Active Your Way: A Guide for Adults” (Department of Health and Human Services, 2008). The second was entitled, “100 Ways to Add 2000 Steps” by the America on the Move Foundation [39]. This brochure suggested 100 ways to increase steps (e.g. Take your dog for a walk) throughout the day. Participants in both groups received the same materials on the same schedule.

#### Message Prompts

Participants in both groups received brief message prompts (≤160 characters) to encourage physical activity. One message was delivered every 9 days over the intervention phase by either email or text message based on a participant's choice. The research team developed a pool of messages that expanded on or complemented messages in the educational components. The prompts were mainly motivational messages, reminders about the health risks of inactivity, benefits of physical activity, and encouraging advice developed by the research team (e.g. Regular physical activity helps prevent type 2 diabetes, heart disease & weight gain. Find time to be active in the next 2 hours!). Participants in both groups received message prompts in the same order on the same schedule.

#### Physical Activity Goals

Physical activity goals were prescribed to both groups, but groups differed on the type of goal received. The Static Intervention group was instructed to meet the goal of at least 10,000 steps each day on at least 5 days per week. Static Intervention participants received this goal on the first intervention day and were reminded of it monthly.

The Adaptive Intervention participants were prescribed new goals each day that adapted to their physical activity. At the end of each day or early the next morning, participants sent their daily cumulative step count obtained from the pedometer to the research team. This brief daily communication was done by email using the subject line only (e.g. Participant #505, 4,351 steps on 4/3/11). This technique was low burden. Once an AI participant sent in their steps for a day, the next step goal was revealed. Goals were good for one day only.

#### Adaptive Goals

**Figure 1:**
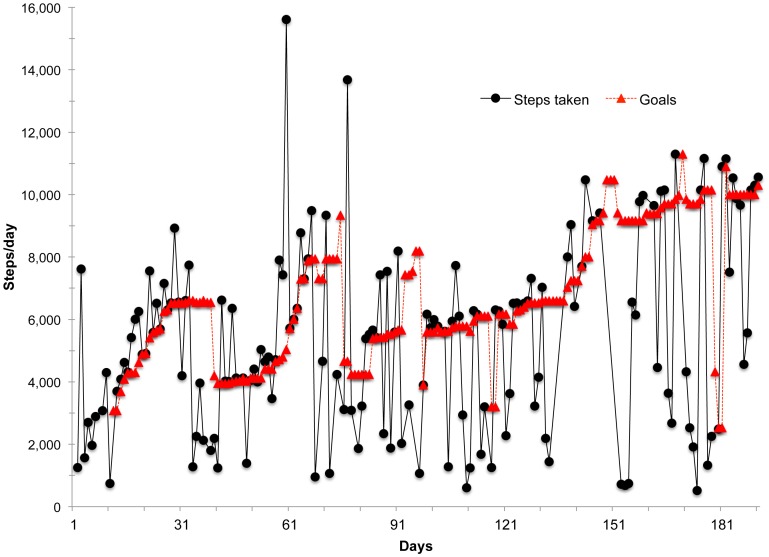
Example of actual steps/day and percentile-based goals over 6 months for a single participant. Figure shows how adaptive goals adjust up and down based on prior performance.

The goal-setting and feedback algorithm was based on a rank-order percentile algorithm derived from recent developments in basic science around schedules of reinforcement [16], [40], [41]. The percentile algorithm requires: 1) continuous and repeated measurements of physical activity, 2) ranking of a sample of behavior (steps/day) from lowest to highest, and 3) calculation of a new goal based on a n^th^ percentile criterion. For example, for one participant, the step count each day for their last 9 days (ranked from lowest to highest) was 1000, 1500, 2600, 4500, 5000, 5700, 6300, 8000, 11,000. The 60^th^ percentile represents a goal of 5700 steps, which becomes the 10^th^ day's goal. The 60^th^ percentile was selected based on previous physical activity research by Adams [40]. Because goals adjusted daily, participants were informed that each new goal was good for only one day. This encouraged participants to email us unprompted daily. Meeting or exceeding this goal would earn praise feedback and a reward point for that day. The 10-day baseline phase was used to calculate the first goal and a moving 9-day window incorporated each new day's steps: newest step count replaces the oldest step count observation. The most recent 9 consecutive days of non-missing observations were used when missing step data was observed during the intervention phase. Complete step data were available for 93.5% of 180 possible days on average. Dead batteries and forgetting to wear the pedometer were main reasons for missing data. It was expected that this algorithm combined with explicit reinforcement procedures would slowly but progressively increase participants' activity over time (i.e., formalized shaping). It is important to highlight that prescribed adaptive goals always fell within each participant's abilities based on a known assessment of their behavior from a moving window of the last 9 days. This is unlike the commonly recommended goal of at least 8,000–10,000 steps 5 days per week, which prescribes a goal that may be beyond current abilities. Figure 1 presents an example of the percentile-based moving-window approach and demonstrates how goals were a function of performance (actual data from one participant).

#### Feedback Messages

Multiple theories indicate that it is critical to reinforce improvements to develop new behavior or strengthen a habit [8], [9], [18], [42]–[44]. The combination of adaptive goals and feedback was expected to provide a strong physical activity shaping program. SI participants received encouraging social feedback (e.g. Well Done! Remember 10,000 steps per day brings you a step closer to good health) for uploading their pedometer steps to Microsoft's HealthVault. For AI participants, once participants sent their steps for the day via email, they received differential feedback messages. On a daily basis, AI participants who did not meet the goal were provided a simple confirmation that steps were entered correctly and provided their next day's goal (e.g. “Steps Received. Goal for 4/1/12 is 4,525 steps”). This avoided negative messages that could be discouraging. Each time an AI participant met his/her goal they received positive feedback in the form of encouragement and praise messages (e.g. “Well done! You're steps closer to good health. Goal for 4/1/12 is 4,525”). We developed a small message pool of 100 statements and a message from the pool was randomly selected each time. Most feedback was sent in less than 2 hours.

#### Feedback Points & Incentives

Once the intervention phase started, SI participants received encouraging escalating financial incentives in the form of gift cards each month for uploading their pedometer steps to Microsoft HealthVault: $5 for month 1, $10 for month 2 and 3, $20 for month 4, $25 for month 5, and $20 for month 6 for a total of $90. AI participants received encouraging feedback and one point worth $1 for accomplishing each step goal. This point system was similar to “credit card reward” points that are exchanged for various items and services [45]. We expected AI participants to meet or exceed an average of 40% of goals over the intervention phase based on the programmed percentile. Participants could not earn or lose points for missing a daily goal or failing to report step counts. Points were exchanged for e-gift cards to several non-food retailers (e.g. Amazon.com, Target.com, iTunes, Barnes and Noble, CVS, American Red Cross), and these were sent every time participants accumulated 5 points ($5, minimum gift card at most companies). AI participants could switch to another store anytime. This structure was implemented to increase immediacy and prevent habituation or satiation to any specific incentive. All incentives for both groups (except for the orientation visit) were provided as electronic gift card codes to various stores. Amounts for the AI group approximated the total amount made available to the SI group to control for cumulative size of rewards.

### Measures

#### Steps per Day

The Omron HJ-720ITC pedometer was the primary measure of physical activity and main outcome. The Omron was small and lightweight with a 7-day LCD display and 41-day internal memory. The HJ-720ITC uses a dual-axis piezo-electric mechanism that counted steps when placed either horizontally or vertically. Participants were instructed to wear the device on their right side. The device was worn via waistband, belt, or kept in a pocket. The Omron has good reliability (CoV<2.1%), is accurate to 3% of actual steps taken, and is less sensitive to error caused by pedometer tilt, which is common among obese individuals [46], [47]. Participants in both groups uploaded the pedometer's 41 day internal memory to Microsoft's HealthVault (www.healthvault.com) every 4 weeks for 6 months over the Internet by connecting the pedometer to their home computer via a USB cable. Microsoft released HealthVault in 2007 as a cloud-based platform to store and share health-related information with other individuals and health care providers. Omron allows Microsoft HealthVault access to their API so the research team could remotely view, access and download participants' pedometer data from the HealthVault website. This ‘off-the-shelf solution’ was free, secure, acceptable to participants, and required little time.

#### Demographics & Socioeconomic Status

Demographic variables collected by survey included: age (years), sex, race/ethnicity (Non-Hispanic White versus Non-White or Hispanic), marital or cohabitation status (married or living together versus other), number of children in household, employment status (full- or part-time employed vs. unemployed), and household income ($25,000 increments ranging from <$25,000 to $100,000 or more).

#### Anthropometrics

Height (cm) and weight (kg) were measured by trained research assistants in triplicate using a research-grade standiometer and scale to estimate body mass index (BMI) (kg/m^2^) at the orientation visit.

### Statistical Approach

Analyses were conducted between 2010 and 2012 with SPSS version 20. Descriptive statistics were examined for all variables. To determine if randomization was successful, statistical comparisons (chi-square, t-tests) between the SI and AI intervention groups were conducted on demographic, socioeconomic, self-reported physical activity, anthropometric variables measured during the orientation visit or baseline phase. Intent-to-treat procedures without imputation were used to preserve random assignment. Average baseline and intervention phase and change scores for steps/day were examined and reported by group, along with an estimate of the between-group effect size (Cohen's d).

Additionally, a random-effects repeated-measures model was used to account for 180 repeated measures (i.e., 6 months x 30 days), two phases, two groups, and nesting of serial observations within participants. Following the model building procedures outlined by Singer and Willett [48], unconditional mean and growth models were specified for steps/day. These first two models served as basic comparison models for more complex model building. Next, time-invariant predictors were added to the mixed-effects repeated measures model including: age, sex, race/ethnicity, marital status and household income. Day of the week (0 = Sunday, 1 = Monday, etc.) was a time varying predictor added to the model to account for any cyclic trends that might occur during the week. Discontinuous change in mean level for each phase was examined by adding a time-varying variable indicating the start of the intervention (0 = baseline, 1 = intervention). A difference in steps/day by group and phase was examined using an interaction term. Discontinuous change in slope was examined independently of the change in the level. Non-linear trajectories were examined and compared to the linear trajectories. A Full maximum likelihood estimation determined population parameter estimates for the fixed effects and variance components. Three model fit criteria identified the final model: 1) the Deviance statistic for nested models and for non-nested models; 2) the Akaike Information Criterion (AIC); and 3) the Bayesian Information Criterion (BIC). Except the unconditional growth model, all models were fitted using a heterogeneous autoregressive error covariance structure to account for autocorrelation.

We also examined goal attainment by group status. Whether participants met their step goal for each of the approximately 170 days of the treatment phase was computed for both groups by comparing participants' step counts to their prescribed goal each day (i.e., 10,000 steps for the SI group and adaptive goals for the AI group). A day's goal was classified as “met” for that day if a participant's step count was greater or equal to their prescribed goal. Additionally, to examine the effect of goal attainment (and reinforcement for the AI group) on future behavior, we examined the influence of meeting a goal or not on the next day's goal attainment and step performance for the 170 treatment phase days for each participant. This estimate compared each day's values (x) to the next day's values (x+1). For example, if a participant met their goal today, we would compare today's step count with that obtained tomorrow (x+1). If x+1≥x, then we classified the next day as an increase or same. If x+1<x, then the following day was classified as a decrease. For goal attainment, if a participant met his/her goal today (x), we would compare to whether the participant met the goal or not tomorrow (x+1). This calculation was done for each participant for all treatment days.

Because the study included intensive repeated measures of step counts and an adaptive intervention, intra-individual plots of variation in steps/day over 180 days are presented. We selected 4 plots (2 from AI participants and 2 from SI participants) to highlight differential patterns across 6 months. Traditionally, published intra-individual variation observed in these figures is masked by aggregated pre/post summary statistics (e.g. means, standard deviations) of groups by phase.

## Results

### Screening and Baselines

**Figure 2:**
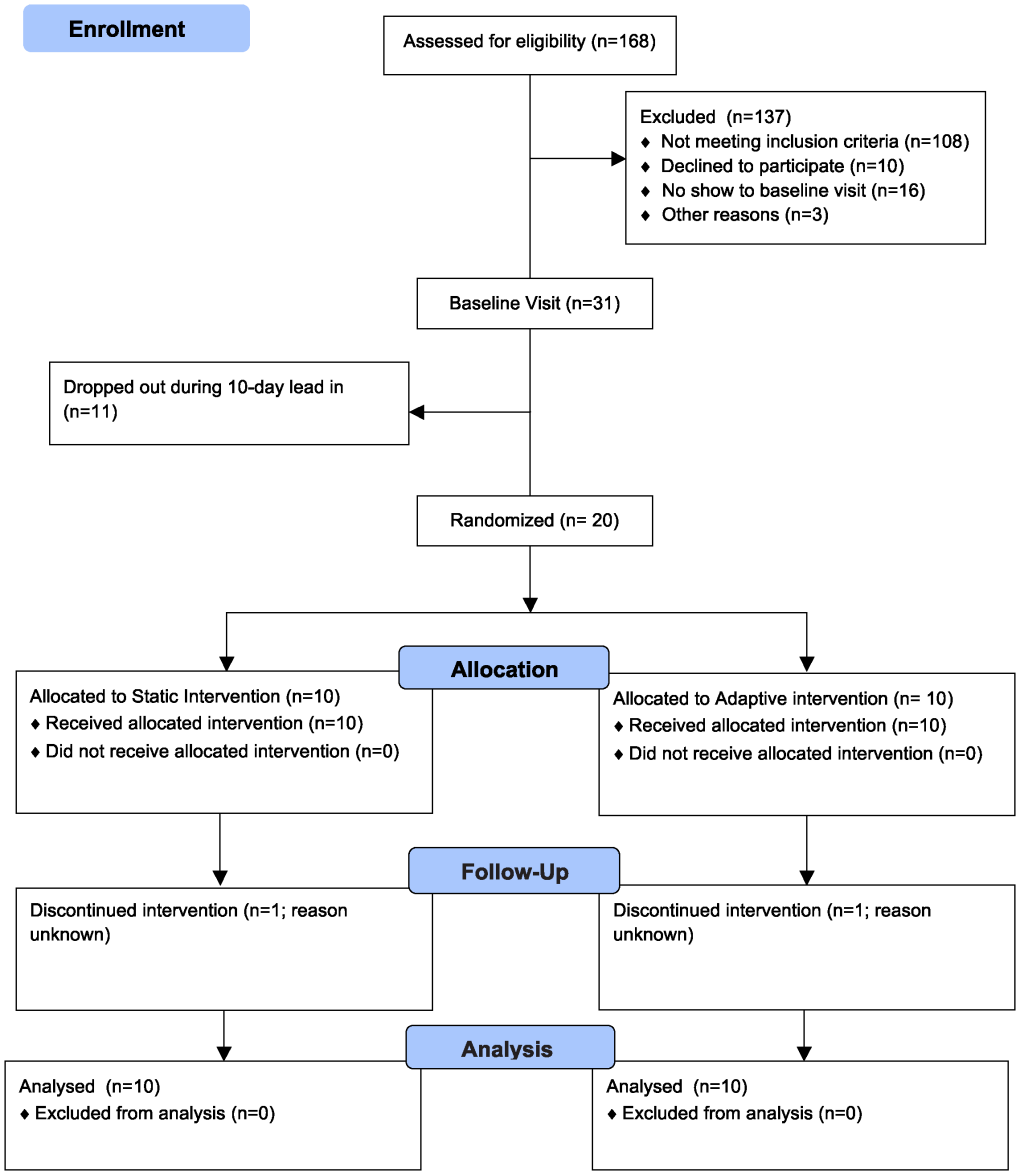
Participant recruitment flowchart.

Figure 2 presents the recruitment process that occurred between April 2010 and May 2011. Recruitment materials resulted in 197 individuals who expressed interest in participating. Of those, 168 were screened and 137 were determined to be ineligible or failed to show up for orientation visit and excluded. Main reasons for ineligibility included: normal BMI or a BMI of 45 or higher (n = 45), too physically active (n = 35), currently on a prescription medication (n = 22), no show (n = 16), declined (n = 10), no computer or internet access (n = 3), currently enrolled in another study (n = 3), health condition (n = 2), or leaving study area (n = 1). All eligible individuals were invited to visit the research office for informed consent and to begin the study; 31 of 47 attended the orientation day appointment (baseline day 0). Twenty of 31 individuals completed the run-in phase, unmasked their pedometers, and uploaded their pedometer data on day 10. Main reasons for not completing the run-in phase were: incompatibility of the pedometer with participants' usual wardrobe (e.g. dresses), dislike of wearing a pedometer, and computer problems.

Table 1 shows no significant differences by group status for demographics, personal characteristics, or anthropometric outcomes. During the blinded baseline phase, the Static Intervention group averaged 5,364 (SD  = 1,145) steps/day and the Adaptive Intervention group averaged 4,555 (SD = 843) steps/day. During the intervention phase, the SI group averaged 6,348 (SD = 671) steps/day and the AI group averaged 6,760 (SD = 1,078) steps/day. This outcome represents a 984 steps/day (18%) improvement for the SI group and a 2,205 step/day (48%) improvement for the AI group; a moderate-to-large between-group effect (Cohen's d = .74) between the two physical activity interventions.

**Table 1:** Demographics and personal characteristics by group.^a^

	Static Intervention	Adaptive Intervention	p-value
	(n = 10)	(n = 10)	
Age	39.27 (10.02)	34.53 (8.14)	.26
% Female	80.0	90.0	.53
% Non-White	60.0	60.0	.65
% Married or living w/sig. other	40.00	60.00	.37
# Children	0.9 (1.29)	0.9 (1.45)	1.0
% Employed	90.00	90.00	1.0
Household income (median)	$50,000 – $74,999	$25,000 – $49,999	.75
Weight (kg)	80.43 (9.38)	80.55 (8.01)	.98
Height (cm)	163.43 (8.43)	164.46 (5.20)	.75
BMI	30.1 (2.16)	29.79 (2.89)	.79

a Values are means (standard deviations) unless noted otherwise.

**Figure 3:**
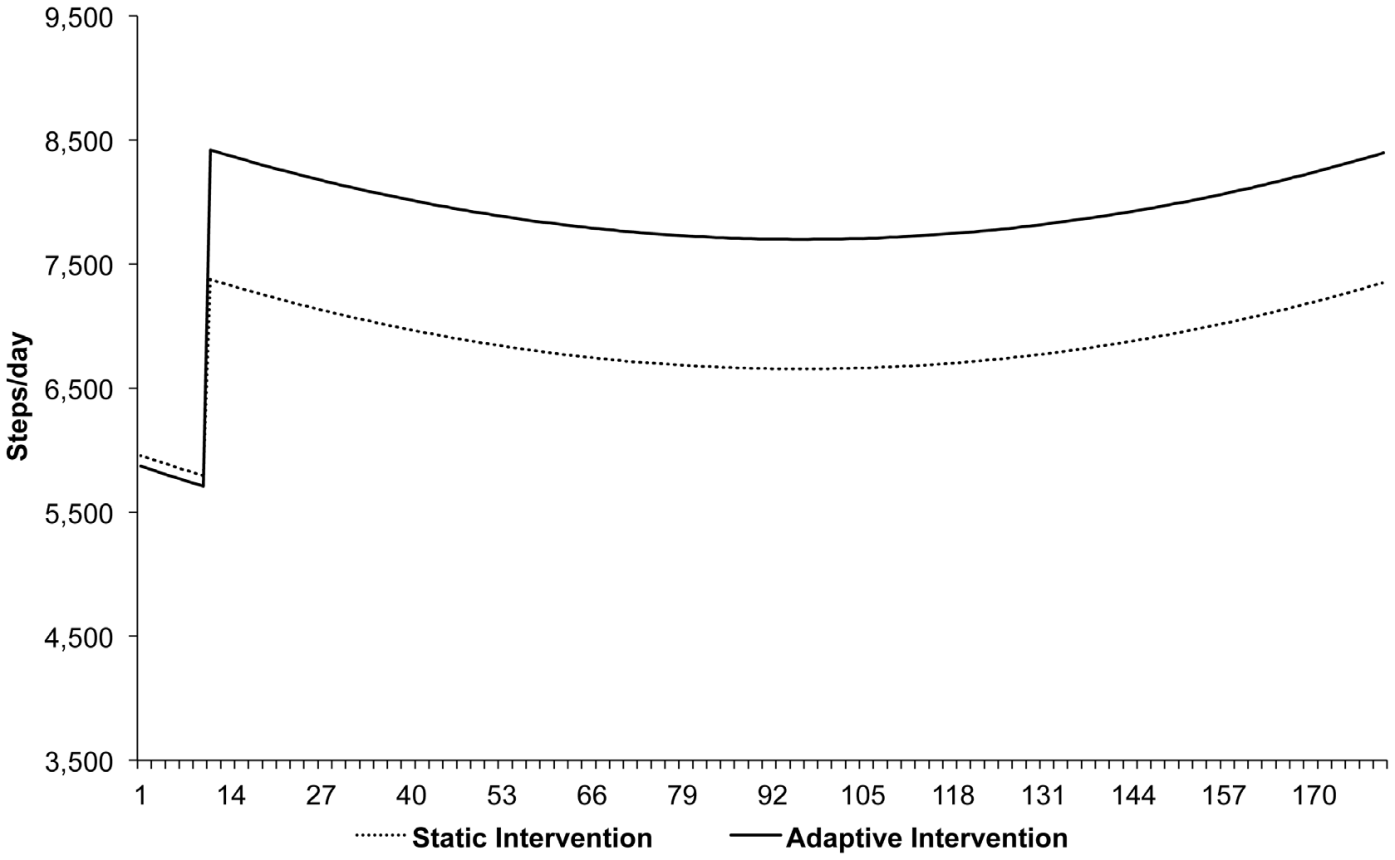
Prototypical trajectories^a^ for Adaptive and Static Intervention groups based on mixed-effects repeated-measures model. ^a^Prototypical trajectories for each group represent values for a 45-year old single women, non-white, with an income between $25,000–$49,000.

Further analyses with 180 repeated measures showed an autocorrelation (.265) requiring a multi-level model. Table 2 shows the final mixed-effects repeated measures model for steps/day accounting for autocorrelation of nested values after adjusting for time, time^2^, sex, age, racial/ethnic group, marital status, household income, and day of the week. The model showed non-significant differences at baseline for all variables. However, after adjusting for demographic and personal characteristics, the AI group had 86 fewer steps/day at baseline (*P* = .93). A significant effect for study phase (*P*<.001) was observed with the treatment phase showing greater steps/day relative to the baseline phase. However, a significant group by study phase interaction was also observed (*P* = .017). Thus, the SI group increased by 1,598 steps/day on average between baseline and treatment phases after adjusting for covariates. The model-adjusted increase for participants in the AI group was 2728 steps/day on average between baseline and treatment phases; a significant between-group difference of 1130 steps/day. Figure 3 displays the AI and SI non-linear trajectories, based on the coefficients obtained from Table 2, for a prototypical participant defined as 45-year old single women, non-white, with an income between $25,000-$49,000.

**Table 2:** Mixed-effects repeated-measures model parameter estimates for steps/day.^a^

Parameter	Estimate	Std. Error	Sig.	95% CI	
				Lower Bound	Upper Bound
Intercept	4968.62	1531.54	.004	−1792.80	8144.43
Time (days)^b^	−19.09	5.13	<.001	−29.16	−9.02
Time Squared (days^2^)^b^	0.10	0.02	<.001	0.05	0.15
Sex: Male (0) vs. Female (1)	−941.28	1535.45	.55	−4142.34	2259.79
Age (Mean centered)	18.55	62.15	.77	−111.12	148.23
Non-White: Non-Hispanic White (0) vs. Non-White (1)	8.12	796.52	.99	−1652.99	1669.22
Marital Status: Single/Divorced (0) vs. Married/Partner (1)	−2035.31	1347.00	.15	−4845.18	774.57
Household Income (per category)	553.05	481.45	.26	−450.71	1556.81
Group Status: Static (0) vs. Adaptive Intervention (1)	−85.53	927.89	.93	−1981.69	1810.62
Study Phase^b^: Baseline (0) vs. Treatment (1)	1597.68	370.04	<.001	872.13	2323.24
Group Status * Study Phase^b^	1130.04	471.62	.017	205.16	2054.92

a Further adjusted for day of the week and specified with random effects for intercept and time and a first-order autoregressive covariance structure with heterogeneous variances. ^b^Time, time^2^, and study phase were time-varying variables.

Table 3 shows goal attainment and its effect on future behavior. There was no between-group difference in the proportion of days participants met the 10,000 steps/day target (AI = 22.6% vs. SI = 22.5%, *P* = .98). However, the AI group was more likely to meet adaptive goals than the SI group was to meet static goals (P<.001). SI participants attained the 10,000 steps/day goal on 22.5% of days on average (range 1.8% – 66.1%) while AI participants attained their adaptive goals 58.2% of days (range 36.1% – 91.0%). The percent of goals attained for the AI group exceeded that set by the percentile algorithm (i.e. 40% for the 60th percentile). On days when participants met a goal, the step count for the following day was higher or the same on a greater proportion of days for the AI group compared to the SI group (AI = 41.1% vs. SI = 23.5% of days).

**Table 3:** Goal Attainment and its Effect on Future Behavior by Group Status.^a^

	Adaptive Intervention Group		Static Intervention Group	
	Goals Not Met	Goals Met	Goals Not Met	Goals Met
Overall Totals	649 (41.8%)	903 (58.2%)	1165 (77.5%)	339 (22.5%)
Next day's goal…^b^				
Met	273 (44.4%)	602 (68.8%)	176 (16.1%)	153 (46.6%)
Not Met	342 (55.6%)	273 (31.2%)	915 (83.9%)	175 (53.4%)
Next day's step count…^b^				
Increased or same	448 (72.8%)	360 (41.1%)	649 (59.5%)	77 (23.5%)
Decreased	167 (27.2%)	515 (58.9%)	442 (40.5%)	251 (76.5%)

a Numerical values equal number of days. ^b^For next day's goals and steps, each day's value (x) was compared to the following day's value (x+1) count. For example, if a participant met their goal today, we would compare today's steps to those obtained tomorrow (x+1). If x+1≥x, then we classified the next day as an increase or same. If x+1<x, then the next day was classified as a decrease. Next day's counts approximate but do not add up to exactly the overall totals because of missing data.

**Figure 4:**
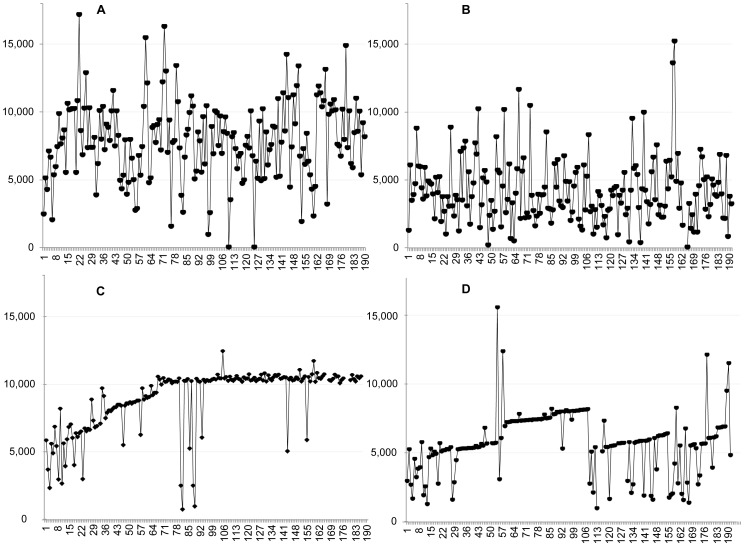
Plots of observed intra-subject variation in steps/day over 6 months for four participants by group. Panels A–D show differences observed in level, trend, and variability on steps/day over 6 months for 4 participants. Panels A and B show participants in the Static Intervention and panels C and D show participants in the Adaptive Intervention. These intra-subject observations are not visible in aggregated group data, but are important discriminations in adaptive interventions.

Figure 4 adds to the overall picture by presenting visual examples of intra-individual steps/day plots for four of the RCT participants over 6 months. Participants A and B were in the SI group (i.e., fixed 10,000 step/day goal, 10,000 steps/day target behavior). Participant A achieved 10,000 steps/day on 28% of the possible days and averaged 8,078 (SD±2,845) steps/day during the treatment phase while Participant B met this goal on 3.1% of possible days and averaged 4,202 (SD±2,364) steps/day during the treatment phase. Both Participants A and B showed patterns with substantial day-to-day variation in steps/day while attempting to reach 10,000 steps/day. Participants C and D were in the AI group (adaptive goals, 10,000 steps/day target behavior), and both met their adaptive goals more than 40% of the time, as designed. Participant C averaged 9,099 (SD±2,099) steps/day while Participant D averaged of 5786 (SD±2231) steps/day during the treatment phase. Unlike the SI group participants, several AI group participants showed a markedly reduced variability and an accelerating trend during the intervention phase.

## Discussion

Prior studies using pedometers and goal setting have asked participants to set weekly goals [22], [26]–[31], prescribed goals for participants by adding standard amounts to baseline levels (e.g. 250 steps/day increase each week) [3], [32], or provided a static goal, such as 10,000 steps/day for the duration of the study [33], [34]. The current pilot study tested a novel approach that prescribed *daily* adaptive goals and feedback based on an algorithm using participants' own behavior in a randomized controlled trial. The difference of 1,130 steps/day between the two physical activity interventions suggests that the multi-component adaptive intervention was efficacious at increasing steps/day relative to a static physical activity intervention also designed to increase steps/day. This comparative study between two types of physical activity interventions suggests that a more intensive, adaptive goal setting and reinforcement approach may be more efficacious than static interventions that focus only on achieving a threshold of 10,000 steps with minimal feedback.

A recent meta-analysis by Conn et al. found that theory-based physical activity interventions resulted in about 15 minutes per week of moderate-to-vigorous activity relative to comparison groups [49]. Norman and colleagues' review of e-Health studies found small effect sizes (ranging from −.03 to .43) for 14 physical activity interventions [50]. Bravata's meta-analysis of pedometer-based interventions found 2,007 steps/day improvement on average compared to baseline with 95% confidence interval of 878 to 3129 steps/day, and the review found that the strongest predictor of improvement was to accomplish a step goal [20]. The consistency across these reviews suggests that our current theories and methods are producing minimal changes to physical activity that do not reliably attain or sustain large effects. Indeed, our static intervention group reflected these observations with a 1,598 increase in steps/day. Riley and colleagues questioned whether theoretical constructs from popular health promotion theories were up to the task for new opportunities to conduct intensive repeated measures and the possibility of feedback loop and shaping interventions [35]. The moderate-to-large between-group effect (Cohen's d = .74) observed in the current study exceeded those found by previous meta-analyses, and may be attributed to the more intensive daily adaptive goals and incentive structure aspects of the shaping intervention, since those two components were not available or differed for the comparison intervention group. However, we acknowledge that larger effect sizes can occur in early and small studies, so this work needs to be replicated.

In the current study, the AI group met their step goals on a greater number of days compared to the SI group. Sidman et al. compared a 10,000 steps/day goal to a personalized goal in a randomized controlled trial [23]. In that study, a personalized goal was developed between the researcher and participants. Sidman et al. reminded participants of the national physical activity recommendation of 30 minutes/day of physical activity and then asked each participant to select a challenging goal of 1,000 to 3,000 steps above their own mean baseline steps/day. Sidman's personalized group met their goals approximately 47% of days, while the 10,000 steps/day group varied in goal attainment by their baseline physical activity level, with those less active (defined as <5500 steps/day) attaining the 10,000 steps/day goal only 23% of the time. While effective, the ability to scale a personalized goal approach defined by Sidman et al. could be laborious and costly to maintain. In the current study, both groups met the ultimate target behavior (10,000 steps/day) an equal proportion of the time (SI = 22.5% vs. AI = 22.6%). However, the adaptive group met their performance-based goals 58.2% of the time on average. The reason for similar proportions meeting the target behavior of 10,000 steps/day may be reflective of a transition state to a sustained habit and the relatively short duration of the study. If goal attainment is expected to be motivating, achieving goals more often should contribute to more sustained physical activity over time. Perhaps adaptive goals are needed to be sensitive to continuous competing circumstances in individuals' lives and local social and environmental contexts. An algorithm-based approach designed to approximate this approach may be more generalizable, time-efficient, and sustainable in perpetuity.

The differences in and changes to intra-individual variability between participants should be highlighted. As can be seen in Figure 4, participants A and B both showed substantial day-to-day variability in their steps/day while attempting to reach the 10,000 step goal, likely reflecting competing “push and pulls” from the intervention vs. responsibilities of daily life. This pattern was common across the static intervention participants. Patterns for participants C and D revealed that even in the presence of competing demands of daily life, goals that adjusted (goals not shown) in response to their daily life events while slowly increasing in demand allowed them to change more consistently to meet these goals. These gradual changes along with more frequent experiences of success may lead to more stable habit formation. The plots also reveal the unique speed of personalization, unlike static goals (e.g. 250 steps/day or 10,000 steps/day). For example, participant C improved early and quickly and the goals adapted rapidly. Participant D changed more slowly and showed a precipitous decrease in steps around day 100, but recovered over the following months. Goals for this participant were slower to increase and adapted downward to account for the precipitous drop, but still supported a positive trajectory. Participant D reported that this drop was the result of an illness that she eventually recovered from. These examples highlight the potential of adaptive interventions to adjust uniquely and non-linearly to continually support physical activity while decreasing the day-to-day vacillations typically known (but not addressed) in physical activity and chronic disease interventions. The combination of a pedometer and adaptive intervention components makes it possible to design highly personalized behavioral medicine interventions.

Financial incentives are controversial. However, use of incentives to promote behavior adoption and maintenance is not just a rhetorical argument, but also a testable empirical question [10], [51], [52]. A special issue of Preventive Medicine devoted to the topic of efficacy and use of financial incentives for a variety of health behaviors and contexts reflects this position [53]. Jeffery's review of the use of financial incentives for weight loss found that the overwhelming majority of incentive structures used relatively delayed relief or avoidance reinforcement methods [54]. The studies operationalized reinforcement by asking participants to deposit differing amounts with the investigators that they could earn back in small amounts (but delayed relative to the goal attainment) if goals were met. The current study designed a positive reinforcement structure, rather than relief or avoidance reinforcement, based on a percentile schedule with rather immediate consequences (e.g. points on a daily basis with gift cards sent every 5 points). Consistent with Operant principles and to a lesser degree Behavioral Economics [10], we rewarded weight-related behavior change (not reductions in weight) and used “smaller-sooner” ($1 daily) rather than “larger-later” payments in the AI group. This amount was effective in our pilot RCT and the total payment was smaller than those for similar studies using delayed payments [51], [52], [55]. Recent corporate wellness programs, such as Virgin's HealthMiles™ [56], government programs such as Medicaid [57], [58], and prevention programs, such as the Diabetes Prevention Program (i.e., DPP Dollars) [59], have paid individuals small amounts for chronic disease-related behavior change. With increased precision for delivering points or payment contingent on target behavior, an engineered or automated shaping approach may be more powerful and generalizable to existing and future programs, managed by health insurance or corporations to make such interventions time-efficient, scalable and sustainable.

### 

#### Methodological Considerations

This study was innovative in several areas: use of a novel adaptive algorithm for promoting physical activity and testing its function in a free-living sample of adults, prescribing daily adaptive goals based on this algorithm, use of positive reinforcement and an incentive structure linked to several types of electronic gift cards to limit the likelihood satiation and habituation to rewards, and finally use of an off-the-shelf Omron pedometer combined with remote access to participants' steps data via Microsoft's HealthVault. Although the small sample size of this study limited the power to identify an interaction between slope and group status across phases, the intensive repeated measures provided sufficient observations (180×20 = 3,600) and power to detect main effects and a group by phase interaction across time after adjusting for autocorrelation and demographics. The adaptive and static interventions were designed as “package” interventions, and the study design cannot disentangle whether the observed effects were due to the adaptive goals or shaping components including reinforcement structure/delivery between groups. However, the two groups were randomized and matched on several intervention aspects such as pedometer reactivity, brief educational materials, message prompts and incentive amounts, so these components can be eliminated as explanations. In our theoretical model, the adaptive goals functioned as stimulus control that signaled the existence of a feedback system that varied in its demand as shaping occurred over time. Additional limitations should be noted. The Omron pedometer's form factor was bulky and women reported that the pedometer could not be worn without being conspicuous when wearing a dress. These two factors explain the majority of attrition during the run-in period. Finally, the sample included English speaking, mainly non-white, inactive, overweight and obese women not using prescription medications with daily access to the Internet. The proportion of women in our sample limits generalizability to men, but this proportion is similar to other physical activity interventions [49]. Future studies are needed to confirm these results in larger, more diverse samples and examine the independent effects of adaptive vs. non-adaptive goals with and without financial incentives.

This study employed a theory-based, adaptive physical activity treatment using pedometers and adaptive goals and feedback to test the intervention among inactive overweight adults compared to typical static goals and feedback. The study showed the potential for personalized behavioral medicine that adapts uniquely to a participant's performance to increase physical activity, potentially producing stronger habit formation, as was observed by decreasing day-to-day variability for several AI group participants. The adaptive algorithm delivered by email is a “behavior change technology” that could be incorporated into m-Health or e-Health technologies for various behaviors and scaled to reach large populations. While this study reported short-term effects, it demonstrated principles and technologies that offer potential to shape physical activity and other behaviors for a population. Future studies can refine the percentile schedule and feedback methods, and the adaptive intervention can be delivered in an engaging manner via mobile applications (i.e., texts, apps) and other automated technologies [60]–[62] to many people, on an ongoing basis, at very low cost.

## Supporting Information

Checklist S1 CONSORT Checklist
(DOC)

Protocol S1 Trial Protocol
(DOC)
