# Supplementary material for: An Adaptive Physical Activity Intervention for Overweight Adults: A Randomized Controlled Trial
Source: PLoS One. 2013 Dec 9;8(12):e82901. doi: 10.1371/journal.pone.0082901 (PMC3857300; doi:10.1371/journal.pone.0082901)
Supplement: Protocol S1 — Trial Protocol (DOC) [file pone.0082901.s002.doc]

# Institutional Review Board

# *Expedited or Full Committee*

**vIRB Instructions**

# Study Abstract

| Provide a brief summary (one paragraph) of your research to include the purpose/objective, methods, subjects, planned analyses, potential benefits, potential risks, and risk management procedures. Please refer to section 4.3 of the IRB Guidebook for guidance on the information to include in this section.  Insufficient physical activity is related to cardiovascular diseases, diabetes, several cancers, and the incidence of overweight and obesity (NIH 1998). The majority of American men and women are insufficiently active. Most physical activity interventions for adults adopt a target behavior that matches national recommendations (e.g. 30 minutes per day, 5 days/week) for all participants. This goal does not consider individuals baseline physical activity level, and therefore such goals may fall outside of an individual’s abilities resulting in individuals never attempting to reach it or failing to reach it. This study will develop and evaluate an adaptive goal setting intervention to promote physical activity behaviors (ActiveRewards Intervention) and compare to a similar physical activity intervention (Comparison Intervention) using a two-group randomized controlled trial design. Participants will include 30 overweight men and women (BMI 25-35 kg/m^2^) between 18 to 55 years. Both groups will receive the following components: 1) a pedometer, 2) self-monitoring of physical activity, 3) brief educational materials, 4) motivational prompts, 5) physical activity goals, and 6) voucher incentives. The ActiveRewards group will receive adaptive goals based on percentiles of a “moving” sample of their recent physical activity, with incentives linked to goal attainment. Comparison intervention participants will receive the “usual care” 10,000 steps per day goal, without linking incentives to goal attainment. The study will compare differences in goal setting procedures that aim to increase physical activity behavior.  •• The aims of this study will be:   1. to determine whether physical activity (pedometer-measured steps/day) in both the ActiveRewards and Comparison groups increased compared to their respective baselines. 2. to evaluate whether the ActiveRewards intervention results in greater change in physical activity (pedometer-measured steps/day) compared to the Comparison group. 3. to assess participants’ satisfaction with the overall program. |
| --- |
| Guidance information is available in the [IRB guidebook](http://gra.sdsu.edu/irb). See the following section(s): [4.3](https://sunspot.sdsu.edu/irb/guidance.jsp?anchor=_Toc65256670) |

# Statement of Purpose and Background

| Discuss relevant background information and literature reviewed to provide the rationale for the proposed research. State the relevance of this research to and potential for contribution to the field of study. Provide justification for involving humans in the research. If relevant, include a summary of pre-clinical/animal data that have been obtained through other research. Please refer to section 4.4 of the IRB Guidebook for guidance on the information to include in this section.  The 1996 Surgeon General’s report on Physical Activity and Health summarized the effect of physical activity (PA) on prevention of morbidity and mortality.^1^ Physical inactivity is associated with an increased risk of cardiovascular mortality, a substantially higher risk of stroke and coronary heart events, and an increase of both systolic and diastolic blood pressures. ^2, 3^ Regular physical activity is protective of some cancers, specifically colon and breast cancers.^4^ The greatest gain in the prevention of all cause mortality appears to occur between inactivity and initiation of moderate intensity physical activity.^5^  Type 2 diabetes is a risk factor for heart disease and stroke and contributes to over 220,000 deaths a year and $132 billion dollars in total healthcare costs.^6^ ^1^ Since 1991, the prevalence of diabetes has increased 61%.^6^ Regular physical activity is protective of developing non-insulin dependent diabetes through improved glucose removal and insulin sensitivity. Additionally, physical activity promotes lean muscle gain and fat loss thereby improving body composition. A dose-response relationship exists between the rate of weight loss and the frequency and duration of activity bouts, and the duration of PA programs, and the addition of physical activity to a diet only weight loss program improves long-term weight maintenance.^1^  The role of PA and overweight and obesity is even stronger. Overweight and obesity have become an epidemic with at least 112,000 premature deaths attributable to inactivity or diet annually.^7^ Overweight-related health problems account for about 5.5% to 7.0% of health expenses in the United States and $139 billion in direct and indirect healthcare costs.^8, 9^ National surveys, using representative samples of the U.S. population, show the prevalence of overweight (BMI = 25.0-29.9 kg/m^2^) and obesity (BMI > 30 kg/m^2^) increased dramatically among adults over the last half century, from 44.8% in 1960 to 66.3% in 2004,^10, 11^ with obesity rates more than doubling over the last 30 years. Sex and age-specific prevalence rates show that between 1960 and 2002 men and women from all age groups gained an average 24 pounds.^12^ Similar prevalence rates are found among other industrialized societies (e.g. Europe), and among urban areas in developing countries.^13^ These findings show that physical activity has protective effects for many diseases, even after controlling for dietary factors.  *Overweight, Energy Expenditure, and Physical Activity*  Because population-level changes in overweight and obesity resulting from environmental selection of genes would take many generations to occur, recent rapid and population specific increases in obesity are likely the result of greater energy intake and reduced physical activity. Hill et al. found that the rise in obesity rates could result from a net increase of only 100-150 calories (kcals) each day.^14^. While a net 150 kcal increase could be a result of increased energy consumption alone, the observed U.S. dietary trends do not support this conclusion. ^15, 16^ A 150 kcal daily net increase is likely to result from a combination of energy balance behaviors, including stagnant or decreasing trends of physical activity. The national recommendations of moderate activity for adults is equivalent to an expenditure of about 150 kcal/day “over and above” energy expenditure from daily activities. ^17^  *Physical Activity Guidelines and Definitions*  National recommendations call for adults to perform 30 minutes of moderate intensity physical activity 5 or more days per week, or 20 minutes of vigorous intensity activity 3 or more days per week, or some combination of both.^18^ This activity can be accumulated continuously or in short bouts of at least 10 minutes periodically throughout the day.^17^ Moderate intensity activity is defined as activity between 3 and 6 METs or equivalently burns 3.5 to 7 calories per minute (kcal/min). ^18^ Examples include brisk walking, bicycling, vacuuming, gardening, or other activities that cause small increases in breathing or heart rate. Vigorous intensity activity is defined as activity equal to or greater than 6 METs or burns more than 7 kcal/ min. ^18^ Examples include running, aerobics, heavy yard work, high-impact aerobic dancing, swimming continuous laps, bicycling uphill or any activities that cause large increases in breathing or heart rate where conversation is difficult or broken. Physical activity can be further distinguished by four broad categories: 1) leisure-time exercise preformed for health reasons (e.g. walking at a health club, running outdoors in evening), 2) daily living activities (e.g. cleaning the house), 3) transportation (e.g. walking to a store or bus stop), 4) occupational (e.g. lifting wood, stair use) activities.^19^ These four categories can include moderate and vigorous activities.  *Behavior Change Theory*  Operant, Social Learning, and Social Cognitive Theories include 3 common behavior change constructs; goal setting, reinforcement, and shaping.^20-22^ However, scientists using these theories vary tremendously on their operationalization of these constructs when designing interventions. For example, goals should be individualized and adaptive to participants’ current behavior repertoire.^23^ Most physical activity interventions adopt a target behavior that match national recommendations (e.g. 30 minutes per day) for all participants and provide reinforcement only when this goal is met. This fixed goal may lie outside of a participant’s current skills and therefore they may never attempt it or fail to reach it. Additionally, scientists may fail to program reinforcement for meeting goal or program poorly by providing incentives or rewards that are not contingent on behavior, too delayed, or infrequent relative to the behavior. Moreover, shaping behavior requires differential reinforcement, which defined as successively reinforcing greater approximations to a target behavior while systematically leaning out rewards for approximations further from the target behavior.^20^ Rarely is this a linear process. This requires researchers to collect repeated measures of behavior and pay attention to small improvements. Adherence to the ideals of reinforcement and shaping constructs is difficult and expensive to meet.  *Pedometer-based Interventions*  Over the last 20 years, the prevalence of people who perform no leisure time PA has remained static at about 25%, even in a context of funding and interventions to change this behavior. Of those adults who are active, the majority do not meet national guidelines.^4^ The most common type of leisure time exercise reported by both men and women is walking, with 44.1% of individuals walking for exercise in the past two weeks.^18^ Individuals report gardening as the second most common activity (29.4%). Unlike gardening, walking is a commonly accepted and highly prevalent activity that can be generalized to most subgroups (i.e. adults, seniors, overweight). Walking may be an ideal exercise behavior to target for public health interventions because it requires no additional training, equipment, or facilities, and can be accumulated through leisure-time, transportation, occupational, or daily activities. ^24^  *Optimal Pedometer Steps per Day.* How many pedometer steps correspond to physical activity guidelines? Hill et al. found that the rise in obesity rates could be a result of a net increase of only 100-150 calories (kcals) each day.^14^ The national recommendation of 30 minutes of moderate activity is equivalent to an expenditure of about 150 kcal/day, “over and above” energy expenditure from daily activities. ^17^ The 10,000 step criteria includes daily activities roughly equivalent to 300-400 kcal/day expenditure, varying based on body size and walking speed. ^17^ One study found that the 8,000 steps/day value was quantitatively linked with the 30 minute recommendation, although generalizability of this value is limited by sample characteristics and size.^25^ A recent review of pedometer-based studies finds that 3,000 to 4,000 steps/day “over and above” daily activities (i.e. 6,000 – 7,000 steps) should approximate the 30-minute recommendations and is in agreement with the 10,000 step/day recommendation.^17^  *Pedometer-based interventions*. A recent systematic review of 26 pedometer-based interventions concluded that the use of pedometers was associated with a 26.9% increase in physical activity.^26^ Randomized controlled trials showed stronger improvements in step counts (i.e. 2,491 steps/day over control group) after weighting by sample size. Observational studies in the review showed similar but slightly lower improvements (i.e. 2,183 steps/day). Improvements of 2,000 steps translates to about a one mile/day improvement. Pedometer interventions have also shown improvements in body mass (0.38 kg/m^2^ reduction compared to baseline) and systolic and diastolic blood pressure (reductions of 3.8 and 0.3 mm Hg).  Bravata et al. also examined the studies for predictors associated with improvements in physical activity. ^26^ Younger participants and ones with lower baseline physical activity levels showed the greatest improvements, although these characteristics were only marginally significant. *The strongest predictor of improvement was having a step/day goal.* Studies without a step goal failed to show statistically significant improvements compared to increases of over 2,000 steps/day for participants in studies with either a fixed 10,000 step/day goal or a more personalized goal. *A comparison of the number of participants who met their goals by goal type (i.e. fixed versus personalized goals) was not possible due to only two studies reporting this critical information.* However, personalized goals typically were based on incremental increases in daily steps over baseline. The authors concluded that the benefits of setting different goals remained unclear. Other predictors of physical activity improvement included participants who kept a step diary and having the intervention in a setting other than the workplace. Pedometer type was not associated with differences between studies.  *Promise of eHealth.*  Technology-based interventions promise to make individual interventions more individualized and easier to disseminate. e-Health can be defined as any interactive technology (e.g., e-mail, Internet, CD-ROM program, handheld computer, kiosk, etc.) used to change behavior.^27^ Interventions that incorporate technology may be able to operationalize theoretically-based behavior change components better than other types of intervention strategies. eHealth interventions may have the ability to maximize a number of theoretically important qualities common to behavior change theories. One possibility of technology is to improve goal setting by assessing smaller milestones more frequently through daily communication, and then, automatically produce new, slightly more challenging goals. For example, Croteau used technology to adjust step count goals biweekly by 5% or 10% to slowly increase daily physical activity to the desired levels, and found large and significant effects over time.^28^ Technology also allows delivery of specific and timely feedback.^29^ Two recent systematic reviews have been published on eHealth interventions related to dietary behaviors and physical activity. Kroeze et al. reviewed studies of computer-tailored materials delivered to participants without person-to-person contact (i.e., by mail, computer, or other media device).^30^ They found little evidence for effective computer-based PA interventions. Norman et al. identified studies for physical activity, dietary behaviors, and the combination of both behaviors.^27^ Norman and colleagues concluded that results were mixed for activity and better research was needed to determine how technology could enhance behavioral outcomes. In particular, the lack of consistency in eHealth intervention outcomes could be explained by a lack of theoretical fidelity or poor study designs.^27^  *Summary*  Regular physical activity is associated with prevention of morbidity and mortality. Yet, a large proportion of the population does not meet the recommended PA guidelines. This may be a result of an imbalance between behavioral consequences for sedentary behavior compared to PA behaviors. Walking is a highly accepted physical activity behavior that all age groups can adopt. Individual-level behavioral interventions have had limited success explaining PA variance and changing PA behaviors. This limited effect may be partially due to a lack of theoretical guidance and intervention theoretical fidelity. eHealth interventions may allow behavioral scientists to improve intervention theoretical fidelity resulting in increased PA among inactive individuals. |
| --- |
| Guidance information is available in the [IRB guidebook](http://gra.sdsu.edu/irb). See the following section(s): [4.4](https://sunspot.sdsu.edu/irb/guidance.jsp?anchor=_Toc65256671) |

# Subjects

Characteristics and Number of Participants

| 1. Describe the characteristics of subjects to be recruited (e.g., number of subjects, gender, age range, etc.). Please refer to section 4.5 of the IRB Guidebook for guidance on describing the subjects that will participate in the research.   b. State how many subjects are planned for recruitment into the study.   c. Describe how the number of subjects was determined. Please refer to section 4.13 to review guidance regarding the relationship between determining sample size and study risks and benefit determination.  A total of 30 overweight (BMI 25-35 kg/m^2^) men and women between 18 and 55 years of age from all racial/ethnic backgrounds will be invited to participate in this pilot study. Because this is a pilot study, the number of subjects was estimated from former pilot studies for physical activity and financial feasibility. |
| --- |
| Guidance information is available in the [IRB guidebook](http://gra.sdsu.edu/irb). See the following section(s): [4.5](https://sunspot.sdsu.edu/irb/guidance.jsp?anchor=_Toc65256672)  [4.13](https://sunspot.sdsu.edu/irb/guidance.jsp?anchor=_Toc65256696) |

Selection Criteria and Participant Screening

| 1. List the criteria for inclusion and exclusion of subjects in this study. Provide a rationale to support the selection criteria.  b. Describe the screening procedures used to determine subject eligibility. Identify how the inclusion and exclusion criteria will be assessed and by whom. For studies that identify specific inclusion and exclusion requirements to determine subject eligibility (e.g., age, physical or psychological condition), append a screening checklist in which specific inclusion and exclusion criteria are listed and defined.  c. Describe how data collected during screening will be handled if the person is found to be ineligible.  d. Describe procedures used to obtain consent from the person in advance of implementing the screening procedures. Please refer to sections 4.8 and 4.9 of the IRB Guidebook for guidance on subject selection and screening procedures.   To qualify as a research study participant, individuals must: 1) live in San Diego County, 2) be between 18 and 55 years old, 3) currently not exceeding 1,000 MET-minutes per week of physical activity determined by the International Physical Activity Questionnaire (IPAQ short form), 4) not suffering from a medical condition or taking medication(s) that would prohibit one from adopting a moderate intensity physical activity program, 5) have a body mass index between 25 and 35 kg/m^2^, 6) not currently pregnant, 7) familiar with email and access to email and the internet daily, 8) not planning to leave San Diego County for more than 10 days over the next 6 months, and 9) not planning to move away from San Diego County in the next 6 months. The telephone screening form will be used to assess whether individuals meet the inclusion criteria. A copy of the screening form has been included with this application  These inclusion and exclusion criteria aim to balance the ideals of recruiting a representative sample and identifying individuals with a high probability of being able to participate and complete the 6 month study. The Physical Activity Readiness Questionnaire (PAR-Q), a physical activity health screener, will assess candidates’ health status for the adoption of an exercise intervention (Thomas, Reading, & Shephard, 1992), and the International Physical Activity Questionnaire (short form) will determine physical inactivity status (Craig et al., 2003). Adults classified as class 2 obese (BMI≥35), older than 55 years of age or using medications that prohibit physical activity will be excluded because of the increased probability that other known and unknown variables could affect the efficacy of the intervention. Such variables can include a higher incidence of cardiovascular diseases, cancers, bone fractures, medication use, death, or other life events.  If a participant is found to be ineligible for the study, their screening information will be kept for up to 1 year. Data from ineligible subjects will be aggregated and used to summarize and compare characteristics to that group who qualified.  We are requesting approval to use verbal consent instead of written consent for the telephone screening of potential subjects. The telephone screening will be used to determine if individuals quality for inclusion into the study. During the telephone screening phone call, participants will be provided a brief overview of the study, informed of the risks involved in answering the screening questions for eligibility, provided telephone number of the PI and SDSU Human Research Protections Program Office, and asked if they would be willing to complete the eligibility screening call. This information is provided on the first page of the telephone script. If an individual qualifies for the study, he or she will be invited to the research office, and, at that time, the study and risks and benefits will be reviewed once again. Written informed consent will be obtained before participation in the 6-month study begins. |
| --- |
| Guidance information is available in the [IRB guidebook](http://gra.sdsu.edu/irb). See the following section(s): [4.8](https://sunspot.sdsu.edu/irb/guidance.jsp?anchor=_Toc65256683)  [4.9](https://sunspot.sdsu.edu/irb/guidance.jsp?anchor=_Toc65256684) |

Special Population

| Vulnerable subjects include children, pregnant women, prisoners, physically or cognitively challenged, economic or socially disadvantaged, subordinate individuals (e.g. students and employees), and fetuses. Additional safeguards for all subjects that are likely to be vulnerable to coercion or undue influence must be included in the study to protect the rights and welfare of these subjects (45 CFR 46.111(7)(b)). Specify what additional safeguards are included to protect the participant. Describe added risks associated with privacy violations and strategies developed to reduce the risk of privacy loss or breech of confidentiality. Provide a rationale for the use of special groups where ability to acquire informed consent may be limited. Please refer to section 4.7 and subsections 4.7.1 through 4.7.7 for guidance on involving vulnerable populations in the research.  No subjects considered vulnerable will be recruited for this study. |
| --- |
| Guidance information is available in the [IRB guidebook](http://gra.sdsu.edu/irb). See the following section(s): [4.7](https://sunspot.sdsu.edu/irb/guidance.jsp?anchor=_Toc65256674) |

Subject Identification

| Describe procedures used to identify potential subjects for recruitment. Please refer to section 4.10 of the IRB Guidebook for information related to subject recruitment. If records are accessed to identify potential subjects, describe procedures used to ensure that records are only accessed by those with legitimate access or who have obtained consent from the individual. Please refer to section 4.10.2 of the IRB Guidebook for information on legitimate access to records  Subjects will be recruited from San Diego County through email announcements, flyers, verbal announcements and electronic listserves posted at various public locations. Posting of these materials may occur in coffee shops, universities and colleges, or electronic websites (e.g. craigslist). These materials are included in our application. |
| --- |
| Guidance information is available in the [IRB guidebook](http://gra.sdsu.edu/irb). See the following section(s): [4.10](https://sunspot.sdsu.edu/irb/guidance.jsp?anchor=_Toc65256685)  [4.10.2](https://sunspot.sdsu.edu/irb/guidance.jsp?anchor=_Toc65256687) |

Recruitment Process

| 1. Identify the location from which subjects will be recruited (e.g., schools, university campus, fitness facilities, hospitals) and confirm that you have obtained permission from the institution(s) to conduct this protocol.   b. Identify how the study will be announced to prospective subjects (e.g., flyers, verbal announcements, paid advertisements, letter, telephone call, public records search, private records search, recruited by a third party (e.g. physician, instructor, employer, etc.) **Attach draft recruitment materials by clicking the "Supporting Documents" link above or in the Protocol Main Menu.** Please refer to section 4.10.1 of the IRB Guidebook for guidance on developing recruitment advertisements.   Drafts of the recruitment materials are attached to this document. Recruitment materials will direct potential participants to call the Active Living Research office. The study coordinator or a research assistant will briefly describe the study to the caller and conduct a telephone screening for eligibility. The telephone screening is expected to take approximately 15-20 minutes. Verbal informed consent will be obtained from potential participants at the beginning of the call. The information collected during the telephone screening will determine whether or not the subject is eligible to participate in the study.  After the initial phone call where individuals are informed of and screen for the study, potential qualified individuals will be invited to schedule an appointment at the research office to review the study, obtain informed consent, and complete the baseline measure. |
| --- |
| Guidance information is available in the [IRB guidebook](http://gra.sdsu.edu/irb). See the following section(s): [4.10.1](https://sunspot.sdsu.edu/irb/guidance.jsp?anchor=_Toc65256686) |

Potential Problems

| Address any potential problems involving subject identification, recruitment or data collection and how these problems will be resolved. Please refer to section 4.12 of the IRB Guidebook for guidance on describing potential problems in your research.  The inclusion criteria are fairly broad and inclusive. The majority of adults do not meet physical activity recommendations. Therefore, major recruitment problems are not expected. Continuous recruitment will occur for several months until 30 participants have been identified. We believe this period of recruitment is a reasonable expectation.  This study aims to help participants improve their activity using several strategies, including the requirement to self-report pedometer step counts each day. Both groups have been designed to increase physical activity and sufficiently intensive to maintain involvement. Participants will be informed at baseline that daily communication is required and should take less than 5 minutes each day. It is expected that some participants will forget to report their step counts occasionally. To plan for this possibility a pedometer with a 7-day memory display will be provided to participants so that they can still email their steps several days later. When participants forget to report their steps, they will prompted via email to report missing information during their next expected communication. Additionally, since rewards will be provided if a participant meets their daily physical activity goal, it is expected that strengthening of their reporting will also occur. These precautions should limit data collection problems.  One concern is that starting a physical activity program might result in injury. We will be screening potential participants for health problems using the PAR-Q and requiring a physician’s waiver to participate in moderate intensity physical activity intervention for those who indicate a health condition. Both interventions are designed to be equal to or less physically intense than national physical activity recommendations, which limits risk to that which would be experienced usually. We will also be advising stretching exercises to maintain a healthy body and providing stretching handout developed by the American Heart Association and the American Council on Exercise (ACE) (**both included in our application**). |
| --- |
| Guidance information is available in the [IRB guidebook](http://gra.sdsu.edu/irb). See the following section(s): [4.12](https://sunspot.sdsu.edu/irb/guidance.jsp?anchor=_Toc65256695) |

# Informed Consent Process and Procedures

Informed Consent Process

| You are responsible for ensuring that the approved consent process is followed. In addition, the participant must sign the IRB-approved consent form before any research activity begins. Approval for your study will be withdrawn if informed consent is not obtained and documented properly. Please refer to section 5.0 and the relevant subsections of the IRB Guidebook for guidance on obtaining informed consent.  a. Describe procedures used to present the study to potential subjects to enhance his/her comprehension of the study information and ability to make a choice or to make an informed decision to participate. Include consideration of the language used to ensure that the information is presented using terms that are clear and understandable and that the setting and timing for explaining the research is conducive to good decision-making. Since informed consent is an ongoing process, include procedures used to inform all research subjects of any new information that might affect their willingness to continue participating in the research. If this study involves a longitudinal design, describe a mechanism whereby consent can be renegotiated, as needed, and subjects can be reminded periodically of the terms of their participation in the research.  b. If minors (children under the age of 18) will be recruited for this study, describe the process used to obtain parental consent as well as assent from the minor child. Please refer to sections 5.6 and 5.7 of the IRB Guidebook for guidance on obtaining parental consent and child assent.   c. If persons who are cognitively impaired will be recruited for this study, describe additional procedures used during the consent process to ensure that the prospective subject understands the information presented about the study. Please refer to section 5.14.2 of the IRB Guidebook for guidance on obtaining consent from cognitively impaired individuals.  The consent form has been submitted with this application for review. Minors and persons with cognitive impairments will not be recruited. Recruitment materials and informed consent documents were written in language at the 6th to 8^th^ grade level. At the baseline office visit, before baseline measures are taken, Dr. Adams or a research assistant will describe the study again, review the study components, and discuss the time commitments and any risks and benefits. Participants will also be informed of their right to refuse to participate at any time. We will solicit and answer any participant questions at this time. Participants will be required to sign and date the consent form indicating that they have given their consent. Participants will be provided copies of the consent form. If new information about the interventions is learned over the course of the study that changes the benefit to harm balance, or confidentiality, participants will be informed of the changes and reminded of their right to refuse to participate. A phone number will be provided on the consent form, so participants can have future questions answered by project staff. |
| --- |
| Guidance information is available in the [IRB guidebook](http://gra.sdsu.edu/irb). See the following section(s): [5.0](https://sunspot.sdsu.edu/irb/guidance.jsp?anchor=_Toc65256742)  [5.6](https://sunspot.sdsu.edu/irb/guidance.jsp?anchor=_Toc65256748)  [5.7](https://sunspot.sdsu.edu/irb/guidance.jsp?anchor=_Toc65256749)  [5.14.2](https://sunspot.sdsu.edu/irb/guidance.jsp?anchor=_Toc65256758) |

Informed Consent Procedures

| 1. Identify who will present the study to potential subjects.   b. Describe the qualifications and training of the person who will be asked to inform potential subjects of the study, answer questions the subject may have about the study and document this process by via a signed consent form.   c. Identify who will verify that the consent form is signed. Please refer to section 5.11 of the IRB guidebook for information related the individual responsible for verifying that the informed consent document is signed.   d. Describe procedures developed to retain the signed copies of the consent document and Research Participant's Bill of Rights (when applicable) in your records for three years.   e. If non-English speaking persons will be recruited to participate, describe the qualifications of the person who will conduct the translated consent process (verbal and written). Please refer to section 5.14.1 of the IRB Guidebook for guidance related to obtaining consent from non-English speaking persons.   f. If a waiver or alteration of the consent process is proposed, provide justification to support this request. Review guidance concerning the waiver or alteration of the consent document in section 5.3, 5.9 and 5.12 of the IRB Guidebook prior to completing this section.   g. If you are requesting that requirements to document informed consent be waived, provide justification to support this request. Review guidance in section 5.10 of the IRB Guidebook concerning waiver of documentation of informed consent prior to completing this section.  h. Attach the consent document(s) by clicking the "Consent Form" link above or in the Protocol Main Menu.   A copy of the signed consent form will be kept at the research office in a locked file cabinet. This document will be retained for 3 years. Training of research assistants will be the responsibility of Dr. Adams. Additionally, any staff who will be communicating with subjects will complete the SDSU Human Subjects Tutorial and have a current certificate on file. Any questions by participants regarding informed consent that a research assistant cannot answer will be directed to Drs. Adams or Sallis. Dr. Adams will be responsible for verifying that the informed consent document was signed. |
| --- |
| Guidance information is available in the [IRB guidebook](http://gra.sdsu.edu/irb). See the following section(s): [5.14.1](https://sunspot.sdsu.edu/irb/guidance.jsp?anchor=_Toc65256757)  [5.3](https://sunspot.sdsu.edu/irb/guidance.jsp?anchor=_Toc65256745)  [5.9](https://sunspot.sdsu.edu/irb/guidance.jsp?anchor=null)  [5.10](https://sunspot.sdsu.edu/irb/guidance.jsp?anchor=_Toc65256752)  [5.12](https://sunspot.sdsu.edu/irb/guidance.jsp?anchor=_Toc65256754) |

# Research Design and Methods

Description of Research Design

| Describe the research design, the scientific rationale underlying the proposed research and the statistical basis for the structure of the investigation. Specify aims of the research that include the hypotheses to be tested, questions to answer, data to be gathered and tested. Describe procedures used to test the hypotheses. Provide enough information about the research design to allow the IRB to make an informed judgment that the design will allow you to answer your questions and produce valid results. This information is critical to the IRB in weighing the potential benefits of the study as compared to the potential risks. Please refer to section 4.13 in the IRB Guidebook for information on IRB review of research design.  *Research Design.*  This study aims to test an adaptive goal setting intervention with frequent reinforcement (ActiveRewards intervention) to increase the number of steps/day to recommended guidelines for inactive adults by comparing to a fixed goal setting physical activity intervention. The study will be conducted with 30 participants using a two-group randomized controlled trial design with repeated pedometer measures over 6 months. The each group will experience two phases during the study consisting of a no-intervention baseline phase and one of two intervention phases. ^31^  *Baseline Phase.* Participants in both groups will be asked to wear a sealed Omron HJ-720ITC pedometer for the first 10 days of the study to collect baseline measures of physical activity. The pedometer will be masked with tape to obscure the number of steps taken. This will be done to reduce participant reactions to the pedometer, which will allow for a shorter baseline phase. Participants will remove the tape seal and report their steps via email after 10 days. After completing the baseline phase, participants will be randomized into either the ActiveReward or Comparison intervention groups.  The intervention components for both groups are shown in the table below. Both groups will receive similar components: 1) a pedometer, 2) self-monitoring of physical activity, 3) brief educational materials, 4) motivational prompts, 5) physical activity goals, and 6) voucher incentives.    *•• Pedometer Assessment.* Participants in both groups will be provided an Omron (HJ-720ITC) pedometer on the first day of the baseline phase, and will continue to use the pedometer throughout the entire study. The HJ-720ITC is small 1 7/8''(w) × 2 7/8''(h) × 5/8''(d) lightweight (less than 1.5 ounces) device and can be worn on a participants’ waistband or belt or kept in their pocket. The device is accurate within 3% of actual steps taken.^32^  *•• Self Monitoring.* Participants will use the pedometer to record the number of steps they accumulate each day. ActiveReward participants will self-report their accumulated steps to the intervention staff each day via email. It is expected to take less than 5 minutes to write an email to report each day. Comparison Group participants will report their steps weekly via email; this should take less than 20 minutes each week. We plan to make reporting easier by requiring participants to report only their ID number, the date, and steps taken. This should limit response burden.  *•• Physical Activity Education Component.* On the first intervention day, participants in both groups will be sent via email two brochures on physical activity. One was entitled, “Be Active Your Way: A Guide for Adults” published by the U.S. Health and Human Services and available at the Centers for Disease Control and Prevention website (www.cdc.gov) (Department of Health and Human Services, 2008). The publication defines physical activity and covers the national recommendations for adults. It also included a number of sections tailored to individuals’ current activity status. These sections included, “Getting Started with Physical Activity”, “Making Physical Activity Part of Your Life”, “Keeping It Up, Stepping It Up” and “Being Active for Life”. The second brochure was entitled, “100 Ways to Add 2000 Steps” by the America on the Move Foundation (www.americaonthemove.org) (America On the Move, 2008). This brochure suggests 100 ways to increase steps throughout the day with the caveat that following only one tip would not provide 2000 additional steps. Tips included “Take your dog for a walk”, “Take an aerobics step class”, “Walk around the outside aisle of the grocery store before shopping”, etc. America on the Move provides these materials free of charge to health professionals and researchers. No further educational materials will be provided to participants. Reviewing these materials is expected to take about 1 hour. The brochures are included with this IRB application.  *•• Motivational prompts.* Participants will receive motivational prompts about their physical activity to help encourage change. It is estimated that one message will be delivered every 2 weeks and participants can chose to receive these via email or text message. The prompts will include motivational messages, reminders about the health risks and benefits of physical activity, and encouraging advice developed by the PIs or research assistants. These prompts may overlap with the messages communicated in the educational component or prompt individuals to re-review the educational brochures. Both groups will receive the same prompts. Reviewing prompt messages is expected to take less than 1 minute each occurrence.  *•• Physical Activity Goals.* Both groups will receive physical activity goals, but groups will differ on the types of goal. The Comparison group will be instructed to try to meet the standard ‘usual care’ goal of at least 10,000 steps each day on at least 5 days per week. This represents a constant and fixed goal to attempt to achieve. As noted in the background selection, this goal approximates the national physical activity recommendation when activities from daily living are included in the count. This goal is commonly heard in the media and has been adopted by physical activity advocacy groups (e.g. America on the Move Foundation). Participants will receive this goal on the first intervention day and will be reminded of it periodically.  ActiveRewards group participants will be prescribed personalized, adaptive goals based on a 10-day moving sample window of their past physical activity measured by the pedometer. Participants will be informed that the goals adapt to their performance. Once a participant emails their pedometer steps, the research staff will send the next day’s step goal via email. Since the goals will adjust daily, each step goal will only good for that day. The percentile algorithm will determine new steps goals.  *•• ActiveRewards Goal Setting Algorithm.* Prior studies using pedometers and goal setting have prescribed goals for participants to meet by adding some progressive amount to participants’ baseline level or have provided a fixed goal, such as 10,000 steps per day, based on guidelines associated with national physical activity recommendations. To our knowledge, no studies to date have applied a continuously adaptive goal-setting algorithm based on participant’s own behavior.  The concept we plan to test is based on percentiles. The Percentiles algorithm requires: 1) continuous and repeated assessments of behavior, 2) rank that sample of behavior from lowest to highest, and 3) apply percentile to that distribution to determine the goal. For example, for one participant, the step count each day for the last 10 days (ranked from lowest to highest) was 1000, 1500, 2600, 4500, 5000, 5700, 6300, 6500, 8000, 11,000. This identifies the distribution of step counts. We plan to use a 70^th^ percentile criterion with a moving 10-day window. Based on this distribution, a goal of 6,300 steps (70^th^ percentile of the distribution) would be provided for the 11^th^ day. Meeting or exceeding this goal would earn a reward voucher for that day. Each new day’s step count will be used to replace the oldest observation of the 10 days, re-rank the distribution, provide a new goal, and slowly but progressively increase participant’s activity behavior over time by always rewarding the top 30% of the distribution of behavior. The algorithm is adaptive, meaning that as physical activity behavior vacillates uniquely for each individual, the algorithm increases or decreases the absolute number of steps so that the goal always represents the best 30% of the last 10 days. Participants will be provided a new goal each day during the intervention phase of the study. **It is important to highlight that prescribed goals will always fall within each participant’s abilities based on a known sample of their behavior from the last 10 days. This is unlike the usual care goal of at least 10,000 steps 5 days per week, which is likely beyond their current abilities.**  One feasibility study to date tested a percentile algorithm for physical activity.^33^ Adams used a 40^th^ percentile and found a 17% increase in steps/day and 32% increase in their moderate-to-vigorous physical activity minutes/day over baseline. At present, we do not know the specific percentile that will produce optimal change for physical activity behavior. Higher percentiles (e.g. 90^th^ percentile) risk the possibility that a participant may never meet the goal since only best 10% of a participant’s behavior would meet it. As found by contingency management studies aimed at decreasing substance use, this could lead to participants experiencing frustration at the difficulty of the behavior change goals, and lead participants to drop out of the study early.^34^ A 5^th^ percentile would be too easy and while this would produce consistent physical activity at some level, it would not result in longer bouts of physical activity.  *** Reward Vouchers.* ActiveReward Group participants will earn one reward point each day they reach their step goal over the 6 months. This incentive system is similar to “credit card membership reward points” that can be exchanged for various items and services. Each point will be worth $1.00. It is expected that participants will meet the programmed average of 30% of goals over the course of the intervention. This equals between $52 and $69 (+10% to be conservative) in incentives over the course of the intervention phase. Participants can bank their vouchers to save for larger payouts (e.g., $10 voucher). Participants will not earn or lose vouchers for missing a daily goal or failing to report step counts. Banked vouchers will never be subtracted. Vouchers will be sent electronically in the form of gift cards that can be exchanged after accumulating a minimum of $5 ($5 is the minimum size gift card at many companies). Electronic vouchers will be sent immediately and will be available for a select number of retailers (e.g. Apple iTunes, Amazon.com) or donation websites (e.g American Red Cross, Kiva.org). Participants in prior studies have requested an option for donation websites.  The Comparison Group will receive $70 in incentives during the intervention phase, but instead of earning for accomplishing physical activity goals, they will receive a progressively increasing amount for exchanging their pedometer each month (pedometers need to be exchanged because they only have 40 days of internal memory). Comparison group participants will a total of $70 on the following schedule: $5 for month 1, $10 for month 2, $10 for month 3, $20 for month 4, $25 for month 5. Both Groups will receive $20 for completing the 6-month questionnaire and returning the pedometer.  Finkelstein et al. found that participants who earned $7.00 for every 1% decrease in body fat lost an average 1.5% of their body weight (or 3 pounds for their 200 pound average participant) by the first opportunity earn the reward at 3 months (Finkelstein et al., 2007). In comparison, the current study offers $1.00 for each instance that participants meet their daily personalized goal which was usually less than 10,000 steps. As noted earlier, 10,000 steps/day is equivalent to 5 miles of walking. A 200 pound individual walking five miles at a moderate intensity (i.e. MET = 3.0) would take about 100 minutes and expend about 455 calories, and would earn $1.00. After matching on calorie expenditure, the current study offers an incentive about 119% more (Finkelstein et al.: 10,500 kcal = $10.50; current study: 10,500 kcal =$23.00) for the same calories expended (assuming a fixed caloric intake). However, the current study will not usually prescribe goals this difficult, so this difference could be greater. Nevertheless, compared to Finkelstein et al., the current study maximizes the fidelity of incentives by: 1) providing opportunities to earn smaller rewards more frequently (i.e. a $1 reward each day versus $7 every 3 months), 2) using a direct contingency of reinforcement for physical activity - instead of weight loss, which can include a number of desired and undesired behaviors, and 3) has a shorter latency between the occurrence of physical activity bouts and the presentation of the reward (i.e. the same day versus 3 months later for the Finkelstein et al. study).  **Statistical Considerations**  Our null hypothesis is that the ActiveRewards Intervention group will not differ in their average steps/day change compared to the Comparison intervention over the course of 6 months. The alternative hypothesis is that ActiveReward participants will show greater improvements in their physical activity change compared to the Comparison Group. Specifically, a mean difference of 1,000 steps/day between groups will be considered practically important. This effect is lower than the mean change observed by a recent systematic review of pedometer interventions but realistic given the pilot nature of the current study .^26^ Because this is a pilot test designed to test the feasibility of using percentiles for promoting exercise, we plan to recruit 30 participants into the study. Thirty individuals is estimated to be sufficient sample to test the concepts and determined to be financial feasible. The pilot study will test the intervention efficacy (i.e., ‘proof of concept’) and will be a preliminary study for future grants to explore this line of research.  **Data Analysis**  **Aim #1.** **To determine whether physical activity (pedometer-measured steps/day) in both the ActiveRewards and Comparison groups increased compared to their respective baseline levels.** We plan to analyze this aim using descriptive and visual methods. Means, standard deviations, and other descriptive data will be summarized by group and intervention phase. Pedometer data will be plotted on a time-series graph and phases delineated to evaluate the change in level, slope, and variability between the baseline and intervention phases of the study for all 30 participants independently and their group means. Regression models may be used to explore change by phase for each group. It is expected that each intervention phase will produce changes in the average number of steps taken per day.    **Aim #2. To evaluate whether the ActiveRewards intervention results in greater change in physical activity (steps/day) relative to the Comparison group.** Since there will be 6 months of daily pedometer data, a multi-level random-effects regression analysis will allow us to detect differences between intervention groups. The independence of observations assumption of many statistical tests are violated with time series data because repeated measures of behavior tend show serial dependence.^35^ The multi-level random-effects analysis will allow us to use all of the available time-unstructured data from the sample of participants without imputing missing values, thus allowing for a smaller sample size. This modeling approach will also allow us to explore the autocorrelation that exists with repeated measures data.^36^ As noted earlier, a difference of 1000 steps/day between groups would be considered a practically important outcome. |
| --- |
| Guidance information is available in the [IRB guidebook](http://gra.sdsu.edu/irb). See the following section(s): [4.13](https://sunspot.sdsu.edu/irb/guidance.jsp?anchor=_Toc65256696) |

Subject Involvement

| Describe the tasks the subject will be asked to complete and indicate the amount of time that the subject will be involved in each aspect of the study. Please refer to section 4.13.1 in the IRB Guidebook for information on IRB review of tasks associated with participation. Include a description of any investigational, experimental, or special procedures that involve the study participant (medical devices, electrical equipment, etc.).  The telephone screening will require about 20-30 minutes to complete.  For participants, only one office visit will be required, which will occur at baseline. The remaining intervention components and measurements will be delivered via email or mail. The baseline assessments are expected to take no longer than 1 ½ hours. During the baseline visit participants will fill out the PAR-Q, will have their height and weight measured, answer surveys about their physical activity (IPAQ), neighborhood (NEWS), media use, and learn to use the pedometer.  A 6-month follow-up evaluation on the intervention modality will be conducted by phone and will take approximately 30 minutes.  Participants will be required to report their steps weekly for the Comparison group and daily for the ActiveRewards Group. This will require about 20 minutes weekly or 5 minutes daily.  Reviewing the education materials should take about 1 hour. Reviewing the motivational prompts should take about 1 minute each instance.  This study will require participants to mail their pedometer back to us each month (6 times total). Participants will be provided postage paid envelopes. This will take approximately 30 minutes each instance. |
| --- |
| Guidance information is available in the [IRB guidebook](http://gra.sdsu.edu/irb). See the following section(s): [4.13.1](https://sunspot.sdsu.edu/irb/guidance.jsp?anchor=_Toc65256697) |

Research Instruments

The IRB is required to review all research instruments such as surveys, interviews or questionnaires planned for use in data collection. The IRB must review the final instruments prior to approving the use of those instruments for data collection. Within this section, provide a brief description of all measures/instruments used for this study (include psychometric properties if available).  You may attach draft versions of study instruments for review if finalized documents are not yet prepared.  **Attach research instruments by clicking the "Supporting Documents" link above or in the Protocol Main Menu.**

• Steps per day. The primary outcome will be pedometer-derived steps per day. The Omron (HJ-720ITC) pedometer is small 1 7/8''(w) × 2 7/8''(h) × 5/8''(d) lightweight (less than 1.5 ounces), with a 40 day internal memory, and can be worn on a participants’ waistband or belt or kept in their pocket. The Omron uses a piezo-electronic mechanism instead of spring-levered arm to measure steps. The piezo-mechanism has shown to be more reliable and accurate for overweight and obese individuals and for measuring slow walking than spring-levered pedometers when both types are compared to direct observation of steps.^37^ The piezo-mechanism is less sensitive to error caused by pedometer tilt, which can result from excessive midsection adipose tissue, and more sensitive to slower walking speeds. Both conditions are likely with inactive and overweight populations. The Omron pedometer is has shown good reliability and is accurate within 3% of actual steps taken.^32^ Step data will be downloaded from the Omron pedometer.

• Body Mass Index (BMI) A research assistant will objectively measure height and weight by stadiometer and digital scale (in light clothing and no shoes), respectively. Each participant will be measured twice at baseline and an average computed. Body mass index will be computed using the following formula: weight (kg) divided by height squared (m^2^).

• Satisfaction with Intervention Modality. At the 6 month intervention, participants will be asked to rate how motivating or burdensome specific study components were to them, and their overall satisfaction with the study. Participants will also asked about their personal experience with the intervention, any side effects or injuries, and their recommendations for improvement. This consumer satisfaction may be helpful for understanding how participants experienced the study, and for improving the intervention (Winett, Moore, & Anderson, 1991).

• Demographic and Socioeconomic (SES) Variables. Demographic variables including sex, ethnicity/race, age, highest educational achievement and household income, will be collected from participants during the phone screening.

• Neighborhood type. Urban form and neighborhood characteristics will be measured using the Neighborhood Environment Walkability Scale (NEWS). The survey includes eight sections measuring self-reported neighborhood residential density, proximity to nonresidential land uses, access to services, street connectivity, walking and cycling facilities, aesthetics, traffic safety, and crime safety. The NEWS has shown good to excellent test-retest reliability and is able to accurately discriminate high and low walkable communities.^38-40^

• Self-reported physical activity. The International Physical Activity Questionnaire (IPAQ) short form will be used to measure physical activity at baseline to determine whether participants perform less than national MVPA recommendations. The IPAQ asks participants to report their frequency and duration of light, moderate, vigorous, walking, and sitting activities over the last 7 days. The IPAQ has shown to be a reliable and valid measure of physical activity with adult populations.^19^

Study Location

| Identify the location and describe the setting where subjects will participate in this research. Address any special considerations associated with recruitment or data collection at the location (e.g. identifying potential subjects, obtaining voluntary participation, confidentiality of data and privacy concerns). For example, if subjects are school children, identify whether class time is used or if children are participating outside of structured class time (address nonparticipating students, supervision of non-participants, procedures used to pull out children/subjects during class time, etc.). Please refer to sections 4.7.1.1 and 4.7.6 for information related to involving students in research during scheduled class time. Please refer to section 4.13.4 of the IRB Guidebook for guidance regarding study location (Note: Please read this section if your research is supported by federal funds and persons not affiliated with SDSU will conduct this study)  Baseline visits will occur at the Active Living Research office in Hillcrest. All activity that participants adopt will be in their free-living environment. No activity will occur at the measurement offices. |
| --- |
| Guidance information is available in the [IRB guidebook](http://gra.sdsu.edu/irb). See the following section(s): [4.7.1.1](https://sunspot.sdsu.edu/irb/guidance.jsp?anchor=_Toc65256676)  [4.7.6](https://sunspot.sdsu.edu/irb/guidance.jsp?anchor=_Toc65256681)  [4.13.4](https://sunspot.sdsu.edu/irb/guidance.jsp?anchor=_Toc65256700) |

# Potential Benefits

| Describe any benefits expected to result from the conduct of this study specific to a. the participant, b. the population from which the subject was drawn, and to c. society/science. Do not include compensation or incentives offered to subjects as a "benefit" to be gained from the research. Please refer to section 4.14 of the IRB Guidebook information about study benefits.  Inactivity is associated with increased disease risk and mortality. Potential immediate benefits may include increased awareness of their physical activity habits, increased knowledge of physical activity strategies for improving their physical activity habits, improved mood, energy balance, and feeling of well-being. Potential long-term benefits for participants include developing a regular physical activity routine, a potential improvement in their weight status or cardiovascular health, and reduced risk of chronic diseases.  The investigators, however, may learn more about 1) methods of increasing physical activity in overweight populations that may have public health implications for preventing disease and mortality, and 2) how to improve the use of goal setting, motivational prompts, and rewards for health behavior change. |
| --- |
| Guidance information is available in the [IRB guidebook](http://gra.sdsu.edu/irb). See the following section(s): [4.14](https://sunspot.sdsu.edu/irb/guidance.jsp?anchor=_Toc65256701) |

# Risk Assessment and Management

| 1. Description of Risks. Describe the potential or known risks associated with participation in this research. Consider and assess the physical, psychological, social, economic and/or legal harm that may result from participation in this study. Discuss whether risks would exceed those likely to be encountered by the individual in their everyday life. Assess whether the risks and inconveniences associated with the research are reasonable in relation to the anticipated benefits to the subjects and in relation to the knowledge that may reasonably be expected to result from this research.  b. Management of Risks. Describe precautions, safeguards and alternatives incorporated into the research activity to reduce or limit the severity, duration and likelihood of harm. If the study activities place the subject at greater than minimal risk for injury, describe what the potential subject will be told during the consent process and describe whether and who will cover treatment for any injury associated with the study.   c. Data Safety Monitoring Board (DSMB). When applicable, discuss the process used to monitor data collected to ensure the safety of subjects (e.g., clinical trial studies). All Phase III randomized clinical trials supported or performed by NCI require monitoring by a DSMB. For more information on DSMBs, please visit the [NCI website](http://deainfo.nci.nih.gov/grantspolicies/datasafety.htm). If this requirement does not pertain to your study, please state 'DSMB - not applicable' in the text box below. Refer to section 4.15.1 of the IRB Guidebook for guidance related to Data and Safety Monitoring Boards.   All research introduces some risk to participants. We believe that the potential risks of this study are minimal to moderate and may be psychological or physical. Participants may experience: 1) anxiety or embarrassment related to one’s personal behaviors related to physical activity habits, answering questions about one’s health or to the measurement process; 2) feelings of inadequacy or embarrassment if unable to succeed at agreed upon activity goals; 3) concern for privacy related to divulging personal information or loss of confidentiality if a computer is accessed by an unauthorized person; 4) physical injuries such as strains or sprains. Judging from our past experience and the current literature, these risks are of relatively low likelihood.   1. Risks of embarrassment or social discomfort will be minimized by fully informing participants of the topics to be discussed and the specific involvement required of them before they agree to participate. Participants will be informed that they may discontinue their involvement at any time, with no impact to the incentives previously received. Staff will be trained to provide a very positive and supportive context for the intervention and assessment. 2. The percentile intervention is designed to specifically to help participants make small, feasible changes in their activity, by prescribing goals that always fall within their previous 10 day sample of activity. These goals can both increase and decrease depending on their activity over the last 10 days. Thus, success at meeting the goals is likely for at least 30% of their attempts. 3. Participants will be told that all responses are confidential. Risks to confidentiality will be minimized by keeping screening forms, informed consent statements and participant data in separate locked file cabinets so that individuals are not easily connected to the study results. When necessary, data will be transferred from prepared forms to electronic files. Participants’ data will be stored on a password-protected computer and identifying information will not be included in the data set. Participants will be told that the SDSU Institutional Review Board will have access to research records. For individuals who do not qualify, their contact information will be destroyed 1 year after participants have been recruited into the study. All participants contact information will be destroyed 1 year after the intervention is completed. We will not re-use or disclose collected health information for other purposes. 4. Encouraging participants to be more physical active by prescribing goals might result in as strains or sprains. It should be noted that the goals participants will be provided will be equal to or less than national recommendations. ActiveReward goals are designed to both increase and decrease based on a moving assessment of their activity using the last 10 days as a window of observation. Physical activity goals for these participants will always be within the observed range of their behavior over the last 10 days. This procedure guarantees that participants will never be provided a goal outside of their abilities.   To minimize the general risk from adopting a more physical active lifestyle, we will screen for health problems with **the Physical Activity Readiness Questionnaire (PAR-Q)** during the baseline assessment. The PAR-Q has been designed to identify the adults for whom physical activity might be inappropriate or those who should have medical advice before starting an activity program. A copy of the PAR-Q has been included in this application. Additionally, we will provide all participants with **the American College of Sports Medicine and American Heart Association tip sheets** on how to minimize risk of injury while being physically active. Examples of the tip sheets have been included in this application. Dr. Adams or a research assistant will administer the PAR-Q and review these preventive tip-sheet practices with participants. All activity that participants adopt will be in their free-living environment. No activity will occur at the measurement offices. |
| --- |
| Guidance information is available in the [IRB guidebook](http://gra.sdsu.edu/irb). See the following section(s): [4.15.1](https://sunspot.sdsu.edu/irb/guidance.jsp?anchor=_Toc65256703) |

# Confidentiality

Confidentiality Procedures

| Describe procedures used to protect subject confidentiality. Identify who will have access to the collected data, where the data will be stored and for how long. Clarify whether the subject's identity is anonymous (no identifiers) or confidential (identifiers are recorded but concealed). Please refer to sections 4.16, 4.16.1 and 4.16.3 in the IRB Guidebook for guidance on maintaining subject confidentiality  As noted above, risks to confidentiality will be minimized by keeping screening forms, informed consent statements and participant data in separate locked file cabinets so that individuals are not easily connected to the study results. When necessary, data will be transferred from prepared forms to electronic files. Participants’ data will be stored on a password-protected computer and identifying information will not be included in the data set; data will be linked by an ID number. Participants will be told that the study investigators and SDSU Institutional Review Board will have access to research records. For individuals who do not qualify, their contact information will be destroyed 1 year after participants have been recruited into the study. All participants contact information will be destroyed 1 year after the intervention is completed. We will not re-use or disclose collected health information for other purposes. |
| --- |
| Guidance information is available in the [IRB guidebook](http://gra.sdsu.edu/irb). See the following section(s): [4.16](https://sunspot.sdsu.edu/irb/guidance.jsp?anchor=_Toc65256705)  [4.16.1](https://sunspot.sdsu.edu/irb/guidance.jsp?anchor=_Toc65256706)  [4.16.3](https://sunspot.sdsu.edu/irb/guidance.jsp?anchor=_Toc65256708) |

Special Considerations for Maintaining Confidentiality

| Please address any of the following points related to maintaining confidentiality that are relevant to your study: Note: If none of these points are relevant to your study, please state "Not Applicable" in the text box below.  a. Reportable Disclosures. Describe limits to confidentiality. Disclose whether and to whom information will be reported. Please refer to section 4.16.2 in the IRB Guidebook for guidance on reportable disclosures.  b. Coding Data for Tracking Purposes. Describe the coding scheme used to track respondents and non-respondents. Provide justification for recording subject identity or using a code that is linked to the subject's identity. Clarify whether the individual's identity is linked to the code and how this information will be used once data collection is complete. Include a description of how coding is used within the consent document. If it is necessary to track information over time, consider using a coding strategy that is not linked to the subject's identify if at all possible. Please refer to section 4.16.3 of the IRB Guidebook for guidance related to coding for tracking purposes.  c. Image and Voice Recording. If the study involves the use of the audio or video recordings, state how the recordings will be used as well as where they will be stored, who will have access, and the duration of storage Include information on how images may be used within the consent document. Use the Video/Audio Release form if the image or recording will be used outside this research study. If you plan to request the participant's permission to use the image for purposes not directly related to this study, complete the Video Image Release Form (link to form). Please refer to section 4.16.4 for guidance related to image and voice recording.   d. Release of Test Results. If the data collected are clinically relevant, discuss whether test results will be made available to the participant or the participant's physician. If information will be released to a physician, provide an amendment to the consent document that authorizes the release of specific data to the subject's physician. Please refer to section 4.16.6 of the IRB Guidebook for guidance on release of test results.   e. Transportation of Data. If data are collected at an off-site location, describe procedures used to ensure that data will be transported in a manner that minimizes risks associated with the inadvertent loss of data. Elaborate on precautions used to prevent data from being lost or stolen.  f. Certificate of Confidentiality. If the research includes disclosure of potentially sensitive or illegal information, describe additional measures used to protect the participant's privacy and confidentiality. Please refer to section 4.16.8 of the IRB Guidebook for information on how to obtain a certificate of confidentiality.  Not applicable. |
| --- |
| Guidance information is available in the [IRB guidebook](http://gra.sdsu.edu/irb). See the following section(s): [4.16.2](https://sunspot.sdsu.edu/irb/guidance.jsp?anchor=_Toc65256707)  [4.16.3](https://sunspot.sdsu.edu/irb/guidance.jsp?anchor=_Toc65256708)  [4.16.4](https://sunspot.sdsu.edu/irb/guidance.jsp?anchor=_Toc65256709)  [4.16.6](https://sunspot.sdsu.edu/irb/guidance.jsp?anchor=_Toc65256711)  [4.16.8](https://sunspot.sdsu.edu/irb/guidance.jsp?anchor=_Toc65256713) |

# Costs

| Describe the costs that the subject may incur as a result of participation (charges for tests, travel, etc.). If this study exceeds minimal risk, state how costs pertaining to any injury incurred due to study participation will be covered and by whom. A study that exceeds minimal risk means that the probability or magnitude of harm or discomfort anticipated in the research are greater in and of themselves than those ordinarily encountered in daily life or during the performance of routine physical or psychological examinations or tests (45 CFR 46.102). Please refer to section 4.17 of the IRB Guidebook for guidance on costs.  We do not believe that this study exceeds minimal risk to participants. Participation in the study does not involve any financial expense other than the cost of transportation to the measurement office. Participants will be provided a baseline incentive to offset this cost. |
| --- |
| Guidance information is available in the [IRB guidebook](http://gra.sdsu.edu/irb). See the following section(s): [4.17](https://sunspot.sdsu.edu/irb/guidance.jsp?anchor=_Toc65256714) |

**Compensation and Incentives**

| **Section Instructions** |
| --- |
| 1. If an incentive payment or compensation is offered, provide a description of the payment structure to include whether the subject is compensated by the number and type of procedures performed, the amount of time involved, or for each sample collected.  b. Indicate whether payment is made with a check, cash or gift certificate.  c. Provide the estimated value of the incentive.  d. Discuss how the value of the incentive offered was determined.  e. If subject confidentiality may be breached by virtue of incentive payment (e.g. the check must be cleared through a bank or through the SDSU Foundation, consider paying subjects in cash, money order, gift certificate or voucher to avoid documentation of the subject's name in association with the study.) Please refer to sections 4.18, 4.18.1, 4.18.2, 4.18.4, 4.18.5 and 4.18.6 of the IRB Guidebook for guidance related to subject compensation.  f. If a lottery system is used as a method of providing an incentive to participants, provide an estimated timeline for when the information about the drawing will occur, how the person will be notified, how many prizes will be offered and the chances for winning one of the prizes (e.g. You have a one in five chance of winning a prize in the drawing.) Also include this information within the consent document. Please refer to sections 4.18.3 of the IRB Guidebook for guidance related to using a lottery system for subject compensation.   Each participant will receive about $105 in total incentives over 6 months. All participants will receive $15 in cash for completing the baseline measurement visit at the Active Living Research office, and will be required to sign an incentive receipt. Both groups will receive approximately of $70 during the intervention phase. ActiveReward participants will earn $1 for each goal met during the intervention phase, with a programmed 40% of goals met on average (.40 X 173 days = $69). Comparison group participant will earn an increasing amount for exchanging the pedometer monthly during the intervention phase on the following schedule: $5 for month 1, $10 for month 2, $10 for month 3, $20 for month 4, $25 for month 5. Both Groups will receive $20 for completing the 6-month questionnaire and returning the pedometer. Baseline incentives will provided in cash. Intervention phase incentives and 6-month incentives will be provided by electronic gift card issued to their participant ID number. Size of the incentives were based on the estimated cost of travel, amount of time required to complete the measures, and the amount that would result in continued study participation. |
| Guidance information is available in the [IRB guidebook](http://gra.sdsu.edu/irb). See the following section(s): [4.18](https://virb.sdsu.edu/irb/guidance.jsp?anchor=_Toc65256715)  [4.18.1](https://virb.sdsu.edu/irb/guidance.jsp?anchor=_Toc65256716)  [4.18.2](https://virb.sdsu.edu/irb/guidance.jsp?anchor=_Toc65256717)  [4.18.4](https://virb.sdsu.edu/irb/guidance.jsp?anchor=_Toc65256719)  [4.18.4](https://virb.sdsu.edu/irb/guidance.jsp?anchor=_Toc65256719)  [4.18.5](https://virb.sdsu.edu/irb/guidance.jsp?anchor=_Toc65256720)  [4.18.6](https://virb.sdsu.edu/irb/guidance.jsp?anchor=_Toc65256721) |

# Investigator Experience

| Provide a brief summary of the investigator's relevant research experience/training (do not include a vitae). If the investigator is a student, also include a summary of the faculty member's experience responsible for supervising the research. Please refer to section 4.19 of the IRB Guidebook for guidance on investigator experience.  Marc A. Adams, Ph.D., M.P.H. adjunct faculty at San Diego State University and a postdoctoral research fellow at UCSD in cardiovascular epidemiology. He received his doctorate in Public Health (Health Behavior Science). He has 8 years of experience working on intervention studies to change socially significant behaviors and has been the principal investigator on two small intervention studies. Dr. Adams will assume overall responsibility for the conduct of the study, including design, recruitment, informed consent procedures, data collection, analysis, manuscript preparation, administrative and operational issues.  James Sallis, Ph.D. is faculty at SDSU in the Psychology Department and is an expert in physical activity behavior and ecological models of behavior change. Dr. Sallis has conducted numerous research studies and will provide specific expertise on the physical activity components of the intervention, and contribute to the research design, measurement of physical activity, data analysis and manuscript production.  Melbourne Hovell, Ph.D., M.P.H. is faculty at SDSU in the Graduate School of Public Health and an expert in applied behavior analysis applied to public health. Dr. Hovell has conducted numerous intervention studies and will provide specific expertise on the use of reinforcement and goal setting, and participant in research design, measurement issues, data analysis and manuscript preparation.  All investigators have completed the SDSU human subjects training tutorial. |
| --- |
| Guidance information is available in the [IRB guidebook](http://gra.sdsu.edu/irb). See the following section(s): [4.19](https://sunspot.sdsu.edu/irb/guidance.jsp?anchor=_Toc65256722) |

# Conflict of Interest

| The IRB considers the investigator's financial interests when evaluating the protection of human subjects. If a financial interest is reported, the IRB will assess the investigator's objectivity in communicating risks, selecting subjects, promoting informed consent, and gathering, analyzing and reporting data. The SDSU Conflict of Interest committee may also review disclosures where a financial interest is reported. Please refer to section 4.20 and 5.8 of the IRB Guidebook (and appropriate subsections) for guidance on conflict of interest. Identify whether you (including your spouse or dependent child) or any person affiliated with the project has any financial interest, financial relationship, governance or administrative affiliation with any entity that is providing funds for or which has rights to intellectual property resulting from this study.  There are no financial conflicts of interest to report. |
| --- |
| Guidance information is available in the [IRB guidebook](http://gra.sdsu.edu/irb). See the following section(s): [4.20](https://sunspot.sdsu.edu/irb/guidance.jsp?anchor=_Toc65256723)  [5.8](https://sunspot.sdsu.edu/irb/guidance.jsp?anchor=_Toc65256750) |

# References

Please provide a list of references to support the information found in the statement of purpose and background section of this protocol.

Recruitment Materials
All recruitment materials, including verbal recruitment scripts, flyers, postcards, brochures, newspaper advertisements, press releases, or postings on the internet must be attached for IRB review. Recruitment materials are reviewed for the accuracy and presentation of information the prospective subject needs to determine their eligibility and interest. This includes the review of content, language, and design. Information should not be misleading to subjects, as such, the use of words that appear neutral as opposed to sensational are encouraged. Attention should be paid to the use of appropriate graphics, font size and format/design, and to accurate spelling and punctuation.

Study Instruments
Any study instrument(s) being used to collect data must be attached. This includes any tests, questionnaires, surveys, or interview guides that have been developed for the purpose of collecting information from the subject. If a standardized instrument(s) will be used, provide a written description of the measure(s) in the Research Instruments section of the protocol. If the final version of the instrument is not yet developed, a draft or sample may be submitted.

Letters of Authorization
If the research involves any institution, business, or organization apart from SDSU, it will be necessary to include a letter from the organization stating support for the research activity. This letter may be scanned and attached as a PDF file or it may be in the form of an electronic message from an authorized representative of the agency or organization. Alternatively, you can mail a hard copy of the letter to the IRB office: 5500 Campanile Drive, MC 1643, San Diego, CA 92182-1643.
